# Disruption of the angiopoietin-like system connects lipid homeostasis and hypothalamic dysfunction in ALS

**DOI:** 10.1186/s12916-026-04749-4

**Published:** 2026-03-03

**Authors:** Sruthi Sankari Krishnamurthy, Adriana Zardini Buzatto, Caley Campkin, Hans-Peter Müller, Diana Wiesner, Jochen Weishaupt, Veronika Klose, Maximilian Wiesenfarth, Zeynep Elmas, Christine Herrmann, Özlem Parlak, Kornelia Günther, Rami Saad, Ulrike Weiland, Luc Dupuis, Albert Ludolph, Liang Li, Jan Kassubek, Johannes Dorst, Francesco Roselli

**Affiliations:** 1 Department of Neurology, Ulm University, Center for Biomedical Research (ZBF), Helmholtzstrasse 8/2, Ulm, 89081 Germany; 2 CEMMA Graduate School, Ulm University, Ulm, 80981 Germany; 3 Department of Biological Sciences, University of Calgary, Calgary, AB T2N 1N4 Canada; 4 The Metabolomics Innovation Centre, University of Alberta, Edmonton, AB T6G 2R3 Canada; 5 German Center for Neurodegenerative Diseases (DZNE), Ulm, 89081 Germany; 6 Université de Strasbourg, Inserm, UMR-S 1329, Strasbourg, 67081 France; 7 Department of Chemistry, University of Alberta, Edmonton, AB T6G 2R3 Canada

**Keywords:** Lipid metabolism, Angiopoietin-like proteins, Lipoprotein lipase, Hypothalamus, Lipidomics

## Abstract

**Background:**

Alterations in lipid metabolism are manifestations of amyotrophic lateral sclerosis (ALS) that contribute to the risk and rate of progression. Blood levels of triglycerides and cholesterol are altered in ALS patients and pre-symptomatic gene carriers, but mechanistic insights into these changes are lacking.

**Methods:**

Serum samples from sporadic ALS patients (*n* = 118), mutated SOD1 and FUS/TARDBP (*n* = 20, 40, 17, respectively) with age and gender-matched controls (*n* = 96) were analysed for alterations in the angiopoietin-like protein (ANGPTL) system using enzyme-linked immunosorbent assays. *SOD1*^G93A^ murine model was studied at pre-symptomatic (P50), early symptomatic (P90), and fully symptomatic (P110) stages, along with their wild-type (WT) littermates for ANGPTLs. Untargeted lipidomics on serum was performed using high-resolution liquid chromatography-mass spectrometry. Further, the involvement of the hypothalamus was studied using hypothalamic volumetry in patients and an antibody array spanning 308 proteins in mice.

**Results:**

We show that mutation-specific patterns of systemic lipid abnormalities appear in ALS and that they correlate with reduced levels of angiopoietin-like proteins 3 and 4. ANGPTL-3/4, in turn, correlates with hypothalamic atrophy but not with corticospinal involvement, as determined by MRI volumetry and diffusion tensor imaging. Lipid phenotype and decreased ANGPTL in humans are recapitulated in two *SOD1* murine ALS models, in which ANGPTL-3, -4, and -8 expression patterns are consistent with the repartitioning of lipid utilisation from muscles to the brown adipose tissue; systemic levels of ANGPTL-3 correlate with hypothalamic neuroinflammation and vascular permeability and with hypothalamic levels of agouti-related protein and neuropeptide Y.

**Conclusions:**

These data provide a molecular mechanism linking peripheral lipid metabolism to the dysfunction of a specific hypothalamic circuit through the mediation of systemic ANGPTL-3 and -4. This finding constitutes a molecularly defined entry point to manipulate lipid metabolism in ALS.

**Supplementary Information:**

The online version contains supplementary material available at 10.1186/s12916-026-04749-4.

## Background

Amyotrophic lateral sclerosis (ALS) is classically conceptualised as a degenerative disease that affects corticospinal neurons in the motor cortex and motoneurons in the spinal cord [1, 2]. More recently, the identification of hypermetabolism and systemic lipid abnormalities in ALS patients and ALS mouse models has suggested that lipid dyshomeostasis may constitute an integral component of ALS pathophysiology [3, 4].

Total cholesterol (TC) and high-density lipoproteins (HDL) are reduced in ALS patients, along with reports of altered levels of triglycerides (TGs) [5–7]. On the other hand, increased levels of TG have been associated with prolonged survival and better overall prognosis [8–10] and higher low-density lipoprotein (LDL) levels correlated with increased ALS risk in Mendelian randomisation studies [10–15] and worse prognosis [16].

The mechanisms linking ALS pathophysiology and lipid dysregulation remain a subject of active investigation [4]. Interventions with high-caloric, fat-rich supplements have been shown to improve biomarkers of neuronal damage [17] and substantially prolong survival and decrease functional decline in the majority of patients, particularly in fast-progressing patients [17].

Recently, the angiopoietin-like system rose to prominence in the control of systemic lipid metabolism. Loss-of-function mutations in angiopoietin-like proteins 3 (ANGPTL-3) and 4 (ANGPTL-4) correlate with a profound decrease in LDL, HDL, and TC levels in human patients [18–21]. ANGPTL downregulation by monoclonal antibodies or antisense oligonucleotides (ASOs) normalises LDL and TG in refractory hyperlipidaemia [22–29]. ANGPTL-3 and −4, in complex with ANGPTL-8, interfere with the activity of lipoprotein lipase (LPL) [30]. Thus, low levels of ANGPTL-3 or −4 result in high activity of LPL and efficient degradation of TG and cholesteryl esters (CE) in circulating lipoproteins. This leads to the extraction of free fatty acids (FA) from circulating lipoproteins towards consumption in tissues, as well as in the enhanced sequestration of cholesterol-rich lipoprotein complexes in the liver [31].

Ever since the first reports of ANGPTL-3 and −4 regulating lipid metabolism appeared [32, 33], various mechanistic studies established that, though circulating ANGPTL-3 levels remain relatively constant [34], ANGPTL-4 and ANGPTL-8 expression levels differ according to the physiological state and the tissue. ANGPTL-4 expression is induced by fasting in both murine and human adipose tissue, leading to the decrease in local LPL activity and the re-direction of lipids towards oxidative tissues such as heart and skeletal muscle [35]. Alternatively, ANGPTL-8 expression is induced by feeding in the liver and adipose tissues [36, 37]. ANGPTL-8 forms complexes with circulating ANGPTL-3 after feeding, which suppresses LPL activity in oxidative tissues, shifting lipids towards adipose tissue [38]. Based on this, an updated model of triglyceride partitioning by ANGPTLs was proposed [39], where ANGPTL-4, induced by fasting in the adipose tissue, suppresses the local LPL activity, thereby driving lipids towards oxidative tissues and circulation. Conversely, high levels of ANGPTL-3–8 complex during feeding direct triglycerides towards adipose tissue for storage, decreasing their levels in oxidative tissues and circulation.

Thus, angiopoietin-like proteins may be posited to contribute to the lipid dysmetabolism observed in ALS, potentially providing mechanistic insights into this ALS phenotype. Here, we investigated the role of ANGPTL-3–4–8 and how they correlate with the lipidome in several ALS mouse models as well as in a cohort of genetic and non-genetic ALS patients.

## Methods

### Clinical cohorts

Serum samples of ALS patients and controls were obtained from the University Hospital, Ulm and authorised by Ulm University Ethical Committee (Approval no. 19/12). Detailed clinical and demographic characteristics of patients are reported in Table 1. Serum was extracted by centrifuging blood samples for 10 min at 2000 g. Serum was collected and stored in polypropylene vials at − 80 °C until further use. For a subset of these patients and additional healthy controls, neuroimaging data (hypothalamic volume and CST-FA) were available. The clinico-demographic features for the same are reported in Additional file 1: Table S1.

**Table 1:** Clinico-demographic characteristics of patients and healthy controls

	Healthy control	sALS	*mSOD1*	*mFUS*	*mTARDBP*	*p* value^b^
Number of patients^a^	96	117	20	14	3	-
Female	50 (52%)	60 (51%)	12 (60%)	5 (36%)	0	0.3
Male	46 (48%)	57 (49%)	8 (40%)	9 (64%)	3 (100%)	
Age at sampling	64 (53, 70)	65 (48, 62)	53 (48, 62)	47 (41, 56)	57 (51, 72)	< 0.001
Age at onset	-	65 (54, 70) (*n* = 106)	50 (43, 61) (*n* = 20)	49 (39, 53) (*n* = 13)	56 (49, 71) (*n* = 3)	< 0.001
ALSFRS-R at sampling	-	42 (36, 44) (*n* = 102)	41 (35, 44) (*n* = 19)	41 (38, 44) (*n* = 13)	39 (37, 39) (*n* = 3)	0.7
BMI at sampling	-	24.7 (22.1, 27.3) (*n* = 100)	25.8 (22.7, 27.6) (*n* = 20)	25.5 (22.9, 26.5) (*n* = 12)	25.7 (24.4, 29.8) (*n* = 3)	0.8
Disease duration (months)	-	11 (6, 18) (*n* = 105)	22 (12, 38) (*n* = 20)	12 (9, 18) (*n* = 12)	10 (9, 21) (*n* = 2)	0.017

Data are mean (quartiles) for skewed data. *ALSFRS-R* amyotrophic lateral sclerosis functional rating scale—revised, *BMI* body mass index; disease duration is the number of months from the date of onset until the date of sampling.

^a^*n* (%), median (IQR)

^b^Kruskal-Wallis rank sum test

### ELISA of ANGPTL-3,4,8 and PCSK9

Enzyme-linked immunosorbent assays were performed to quantify the concentrations of ANGPTL-3, −4, −8 and PCSK9 in the serum of patients and controls. Human Angiopoietin-like 3 DuoSet ELISA, Human Angiopoietin-like 4 DuoSet ELISA, and Human Proprotein Convertase 9/PCSK9 DuoSet ELISA were used according to the manufacturer’s instructions (R&D Systems, USA). Briefly, 100 µl diluted serum samples and standards were incubated for 2 h at room temperature (RT) on a 96-well plate coated overnight with the capture antibody. The plate was then washed and incubated with the detection antibody for 2 h, with subsequent additions of streptavidin-HRP, substrate, and stop solution. The optical density was measured in ELx800 Microplate Reader (BioTek) at 450 nm with plate correction set to 570 nm, and concentrations were calculated according to the standard curve. Samples were diluted in the reagent diluent at 50 × for ANGPTL-3; 3 × , 5 × , or 10 × optimally for ANGPTL-4; and 100 × for PCSK9 ELISAs. ANGPTL-8 was measured using the Human Angiopoietin-like protein 8/Betatrophin ELISA Kit (Novus Biologicals, USA) following the manufacturer’s protocol. In short, 100 µl of standard and diluted (5 ×) serum samples were incubated on a pre-coated 96-well plate for 90 min at 37 °C, followed by the addition of biotinylated detection antibody, HRP conjugate, substrate, and stop solutions. Absorbance at 450 nm was measured in ELx800 Microplate Reader (BioTek), and concentrations were calculated according to the standard curve.

### LPL activity assay

The Lipoprotein Lipase (LPL) Activity Assay Kit from Cell Biolabs, USA, was used to quantify LPL activity in serum fluorometrically. One hundred microlitres of diluted (100 ×) LPL samples and standards were added to a 96-well fluorescence microtitre plate, along with 100 µl of LPL fluorometric substrate. The plate was incubated for 30 min at 37 °C, protected from light. It was then incubated with the stop solution at RT for 15 min, followed by measurement with a fluorescence microplate reader equipped for excitation in the 480–485 nm range and emission in the 515–525 nm range. The LPL activity within samples was calculated by comparing sample fluorescence to the standard curve.

### Measurement of cholesterol

Total cholesterol, cholesterol ester, and free cholesterol were measured colourimetrically using the Cholesterol/Cholesteryl Ester Assay Kit (Abcam, UK). Briefly, serum samples (diluted 10 ×) and standards were added to the wells, followed by total cholesterol reaction mix (with esterase) or free cholesterol reaction mix (without esterase), incubated for 60 min at 37 °C and analysed with a microplate reader set to 570 nm. Total and free cholesterol were quantified from the standard curve, and cholesterol was determined by subtracting the value of free cholesterol from the total.

### Hypothalamic volume and CST-FA quantifications

#### MRI acquisition and hypothalamus volumetry

T1-weighted 3-D magnetic resonance imaging (MRI) data for hypothalamic volumetry were acquired at a 1.5-T clinical MRI scanner (Symphony, Siemens Medical, Erlangen, Germany) by an MPRAGE sequence with 144 sagittal slices, no gap, 1.0 × 1.0 mm^2^ in-plane voxel size, slice thickness 1.2 mm, 256 × 192 × 256 matrix, TE = 4.2 ms, TR = 1600 ms. Additionally, a diffusion tensor imaging (DTI) study protocol was applied, consisting of 52 volumes (64 slices, 128 × 128 pixels, slice thickness 2.8 mm, in-plane pixel size 2.0 mm × 2.0 mm), representing 48 gradient directions (b = 1000 s/mm^2^) and four scans with b = 0; TE and TR were 95 ms and 8000 ms. For the determination of the volume of the hypothalamus, the T1w data were used for manual delineation of the hypothalamus in coronal planes using a well-established landmark-based procedure within the Tensor Imaging and Fibre Tracking (*TIFT*) software as previously described [40, 41]. In short, a four-step processing pipeline was used that included:

1. Rigid brain transformation along anterior commissure (AC)–posterior commissure (PC) axis
2. Spatial upsampling into a study-specific grid (in-plane resolution of 62.5 × 62.5 µm^2^, coronal slice thickness of 0.5 mm) to improve the accuracy in identifying landmarks and hypothalamic borders
3. Delineation of the left- and right-hemispheric hypothalamus by an intensity-threshold-based semi-manual slice wise identification in coronal slices
4. Normalisation of the hypothalamic volumes to intracranial head volume (ICV)

#### Microstructural MRI analysis

The DTI analysis software Tensor Imaging and Fibre Tracking [42] was used for *microstructural MRI analysis*; the algorithms have been previously described in detail [43]. In brief, after correction for eddy currents and a standardised quality control protocol, stereotaxic normalisation to the Montreal Neurological Institute (MNI) space was performed iteratively using study-specific templates. To map white matter microstructure from the stereotaxically normalised DTI, data sets of all subjects’ fractional anisotropy (FA) maps were calculated. A Gaussian filter of 8 mm full width at half maximum was applied to smooth FA maps for a good balance between sensitivity and specificity. Finally, FA maps were corrected for the covariate age. The corticospinal tract (CST) was identified by fibre tracking on a group averaged data set of controls by tractwise fractional anisotropy statistics (TFAS) [42, 43]; voxels with FA values below 0.2 were not considered for statistical comparison, since cortical grey matter shows FA values up to 0.2.

### Experimental animals

Male wild-type (WT) mice—B6SJLF1 and ALS transgenic mouse model—B6SJL-Tg(SOD1*G93A)*1Gur/J* were used in the study. All experiments have been approved by Tierforschungszentrum Ulm and by the Regierungspräsidium Tübingen under licence numbers 1440 and o.217–9. The mice were locally bred under standard housing conditions, with ad libitum access to water and food and a day-night cycle of 12 h. Only male mice were used in the current study.

### Mouse ELISA assay

Mice were sacrificed at postnatal stages P50, P90, and P110, corresponding to the pre-symptomatic, early symptomatic, and fully symptomatic stages of ALS. Blood was collected in EDTA-coated tubes and centrifuged at 1500 g at 4 °C for 10 min. The resulting supernatant collected (plasma) was immediately frozen at − 80 °C. ANGPTL-3 was measured in plasma using a Mouse Angiopoietin-like 3 Quantikine ELISA Kit according to instructions provided (R&D Systems, USA). Briefly, 100 µl of diluted plasma (100 ×) and standards were incubated for 2 h at RT on the manufacturer-coated plate, followed by adding the conjugate, substrate, and stop solutions. Absorbance was then measured at 450 nm with plate correction at 570 nm, and the concentrations were calculated from the standard curve. The ELISA Kit for Angiopoietin-like Protein 4 (Cloud-Clone Corp, USA) was used to calculate the concentration of ANGPTL-4 in mouse plasma. Shortly, diluted samples (10 ×) and standards were added to the pre-coated wells for 1 h at 37 °C. Subsequently, detection reagents A and B, substrate, and stop solutions were added, and optical density was read immediately at 450 nm.

### Measurement of LPL activity, cholesterol, and triglycerides

LPL activity, total cholesterol, free cholesterol, and cholesterol ester were quantified in the mouse plasma as described above. In addition, triglycerides in the plasma were measured using the Triglyceride Assay Kit—Quantification (Abcam, UK). Standards and diluted samples (5 ×), lipase, and triglyceride reaction mix were added to a 96-well plate and incubated for 60 min at RT protected from light. The optical density was then read at 450 nm.

### RNA extraction and qPCR

WT and *SOD1* mice were sacrificed at P50, P90, and P110, and the following fresh frozen tissues were harvested from them: liver, kidney, white adipose tissue, gastrocnemius muscle, brown adipose tissue, hypothalamus, and cortex. They were used for further RNA isolation, quantitative PCR processing, tissue sectioning, and immunofluorescence staining.

RNA extraction and qPCR were done as detailed [44]. Briefly, to 25 mg of tissue, 1000 µl QIAzol Lysis Reagent (Qiagen) was added and lysed for 50 Hz, 1 min in a tissue lyser homogeniser (Qiagen). The tube containing the homogenate was placed at RT for 5 min. 0.35 ml chloroform was added, and the tube was vortexed for 15 s. After incubation at RT for 10 min and further centrifugation at 4 °C, 12,000 × g for 15 min, the upper aqueous phase containing the RNA was precipitated with 250 µl isopropanol. After 10-min incubation at RT, the tube was centrifuged at 4 °C, 12,000 × g for 10 min. One millilitre of 75% ethanol was added to the pellet, the tube was centrifuged at 4 °C, 8000 × g for 10 min, and the RNA pellet was air-dried, before it was finally dissolved in 15–25 µl of RNase-free water. The concentration and quality were detected by NanoDrop 1000 (Thermo Scientific). Reverse transcription was performed as described previously [45]. Briefly, 1 µg RNA diluted with RNase-free water and 5 µl of random hexamer were incubated for 10 min at 70 °C. Master mix, containing 12 µl RT 5 × buffer, 2 µl dNTPs, 0.5 µl RiboLock Rnase Inhibitor (Thermo Scientific), and 0.5 µl M-MLV RT RNase (Promega), was added. After incubating for 10 min at RT, 45 min at 42 °C, and 99 °C for 5 min, the samples were placed on ice to stop the reaction. The cDNA was stored at − 20 °C and used for qPCR. Two microlitres cDNA was mixed with 5 µl TB Green Premix Ex Taq (Takara Biosciences) and 3 µl primer mix (Additional file 1: Table S2) in a 96-well plate and measured with a Light Cycler 480 II (Roche), with the housekeeping gene GAPDH as a control. The cycle threshold values obtained from the light cycler were used to calculate relative mRNA expression with the 2 − ΔΔCt formula.

### Tissue sectioning and immunohistology

Immunohistology was done as detailed [46]. Frozen brown adipose tissue was embedded in optimal cutting temperature (OCT) (Tissue-Tek, The Netherlands), and 10-µm sections were cut with the cryostat (Leica, CM1950) and mounted on glass slides, followed by a 10-min fixation in 4% PFA. Target retrieval was performed in sodium citrate buffer pH 8.5, followed by blocking the sections in blocking buffer (3% BSA, 0.3% Triton X-100; PBS) for 2 h at RT. The primary antibodies (for an overview of antibodies used, see Additional file 1: Table S3) were diluted in a blocking buffer and incubated for 48 h at 4 °C, followed by 3 × 30 min washes in PBS at RT. The secondary antibodies were diluted in blocking buffer and incubated for 2 h at RT, followed by 3 × 30 min washes in PBS, and the sections were mounted using Fluorogold prolong antifade mounting medium (Invitrogen, Germany).

### Protein extraction

Fresh frozen tissues of the hypothalamus and brown adipose tissue were lysed in 4 × the volume of protein extraction buffer—RIPA buffer (Sigma Aldrich) containing phosphatase and protease inhibitors. The lysate was sonicated for 2-s cycles at 50 kHz, incubated for 30 min on ice, and then centrifuged at 10,000 g for 15 min at 4 °C. The supernatant containing protein extract was transferred to a new tube and stored at − 80 °C until further use.

### Mouse L-series array

Mouse L-308 array (glass slide) was used on WT and SOD hypothalamic protein lysates aged P110. The procedure was performed as specified on the manufacturer’s website (Raybiotech, USA). An equal amount of protein lysate (30 µg) per sample was labelled with biotin. The glass slide containing target spots was blocked, and 40 × diluted biotinylated samples were incubated overnight at 4 °C on a rocking platform shaker. The array slides were washed, incubated for 30 min with 1 × Cy5 conjugated streptavidin (Invitrogen), and rewashed. The completely dried glass slides were then scanned using a GenePix 4000B array scanner (Molecular Devices, LLC) to obtain the foreground and background intensities.

### Microscopy and image analysis

Fluorescent images were acquired as detailed in [47] with a Leica CM1950 (Germany) confocal microscope, with a 20 × oil-immersion objective and 1.5 added optical zoom; optical section thickness was set at 200 nm; and images were acquired at 12-bit depth. Imaging parameters were kept constant across replicates and genotypes. Ten optical sections were acquired for each section. Image quantification was performed in ImageJ (National Institutes of Health). For the evaluation of the intensity of ANGPTL-4, all optical sections were collapsed in maximum-intensity projection, subjected to rolling-ball background subtraction, thresholded, and the mean fluorescence intensity per cell was logged.

### Protein array analysis

The genepix settings file pertaining to each spot in the slide was obtained after overlaying the Genepix array list (GAL) file to the raw TIFF images of the protein arrays in GenePix Pro 7.0 to generate GenePix Result (GPR) files. The intensities obtained from each sample’s gene pix result file were loaded onto a protein array analysis pipeline [48]. Background correction was performed using the “normexp” backgroundCorrect function. Subsequently, data were normalised to account for technical variation between arrays. For global normalisation, intensities were normalised using the normalizeBetweenArrays function with the cyclicloess method. Additionally, the batch effects were compensated using the ComBat function. After normalisation, spot replicates were aggregated by calculating the mean of replicate intensities for each protein, yielding a single value per protein per sample. Differential protein abundance was assessed using Bayes technique, a linear model implemented in the limma package for differential expression analysis. Multiple testing correction was applied using the Benjamini–Hochberg false discovery rate (FDR) method to control the false discovery rate. Proteins with an adjusted *p* value < 0.05 were considered statistically significant in WT vs SOD1 comparisons. Additionally, Spearman rank correlation was performed for the proteins in the antibody array against the systemic concentration of ANGPTL-3,4. Targets with an *R* value above 0.35 and FDR-adjusted *p* value < 0.10 were considered statistically significant and considered for a gene ontology analysis. This analysis was conducted using the STRING-db online platform available at string-db.org. The list of proteins was uploaded to STRING-db and *Mus musculus* was selected. A background list consisting of all the 308 proteins in the array was provided to the analysis to ensure accurate background correction. No specific interaction score cutoff was applied for the GO enrichment step. GO enrichment was performed by employing a hypergeometric test with Benjamini–Hochberg false discovery rate (FDR) correction for multiple comparisons. GO terms with an adjusted *p* value (FDR) < 0.05 were considered significantly enriched.

### Lipidomics

Mouse plasma and human serum samples were shipped to The Metabolomics Innovation Centre (TMIC, Edmonton, Canada) for untargeted lipidomics. The mouse dataset included 92 samples from 40 wild-type and 52 *SOD1* animals, while the human cohort was composed of 136 samples from 40 control volunteers and 94 ALS patients, including 37 sporadic ALS cases, 20 *SOD1* mutations, 14 *FUS*, and 3 *TARDBP* cases. The mouse and human cohorts were treated separately for sample preparation, analysis, data processing, and statistics. Samples were randomly split into batches of 6–13 for preparation and analysis (8 for mouse and 11 for human samples). The detailed analysis method is available as previously reported [49–51]. Briefly, each aliquot of 6.0 µl of plasma or serum was mixed with 15 deuterated lipid standards and extracted with a modified Folch method (liquid–liquid extraction with 8:4:3 dichloromethane/methanol/water) using a 40:1 sample/solvent ratio. The organic phase was evaporated to dryness and resuspended in chromatographic mobile phases for reversed-phase LC–MS/MS analysis using a Thermo Vanquish LC with a C18 column coupled to a Bruker Impact II QToF in positive and negative ionisation. The acquired chromatograms were processed with NovaMT LipidScreener (Nova Medica Testing, Edmonton, Canada) [52, 53], an in-house developed, Python-based software solution explicitly designed for the TMIC untargeted lipidomics platform (proprietary code), including peak picking, alignment, data cleansing, polarity merging, lipid identification, and normalisation. Features detected for ≥ 80% of the injections in at least one sample group were kept for further processing. Lipids were identified by *tandem*-MS spectral match through comparisons with literature databases, such as MSDial LipidBlast, and m/z match. All identifications had a m/z tolerance of 5.0 mDa or 6.0 ppm and passed through a filtering and scoring approach based on the expected analytical performance of each lipid species (e.g. retention time, adduct pattern, ionisation performance) and biological intelligence [54] (the expected presence of each lipid species in mammalian samples). The identifications were categorised into three tiers. Tier 1 corresponds to high-confidence MS/MS spectral matches with a similarity score higher than 500 (within a 0 to 1000 scale where 1000 corresponds to a perfect match), while tier 2 IDs have scores between 100 and 500. Lipids identified in tier 3 are low-confidence m/z matches. Lipid classification and shorthand notations followed the guidance of LipidMaps [54]. The peak intensities (i.e. peak height) of identified lipid species were normalised by the most similar internal standard amongst the 15 deuterated lipids added to each sample before extraction. Peaks with a relative standard deviation higher than 30% for quality control experimental replicates (i.e. low reproducibility) were removed from the dataset. The intensity ratios (i.e. the intensity of the lipid divided by the intensity of the most similar internal standard) were further normalised to the median within each sample and auto scaled for statistical analysis.

### Statistical analyses

For ELISAs and bioassays, all values are provided as mean ± SEM. Box-and-whiskers plots are defined such that the boxes extend from the lower to upper quartile values, with a thick line at the median. *, *p* < 0.05; **, *p* < 0.01; ***, *p* < 0.001; and ****, *p* < 0.0001. Statistical tests were performed in R (version 4.2.2), and figures were generated using R (version 4.2.2) and ImageJ (version 1.54).

All multivariate analyses were performed in R (version 4.2.2). For demographic and clinical variables, relevant columns were selected from the datasets and descriptive statistics were calculated for continuous variables as mean ± standard deviation, and categorical variables as counts and percentages. Comparisons between groups were performed using the gtsummary package in R, applying *p* values to assess statistically significant differences. To assess the influence of demographic and clinical variables on the lipidomic profiles, multivariate analysis of variance (MANOVA) was performed using the top 10 principal components (PCs) as dependent variables. Predictor variables included group, age (sampling), age (onset), sex, BMI, and ALSFRS-R, with interaction terms between group and the covariates to evaluate potential group-specific effects. The Pillai’s trace test was used to determine multivariate significance due to its robustness to unequal sample sizes and correlation amongst dependent variables. MANOVA results were interpreted to identify the effect of each factor and interaction on the variation captured by the principal components.

For lipidomics, uni- and multivariate statistical analysis was performed with NovaMT LipidScreener and MetaboAnalyst 5.0 (www.metaboanalyst.ca/) using the median and internal standard-normalised intensity ratios for annotated lipids (tiers 1, 2, and 3). Non-informative compounds (internal standards, common contaminants, and features with low experimental reproducibility) were filtered out. Univariate statistics were performed by calculating fold changes (FC, ratios between normalised peak intensities) and *t*-tests (LipidScreener: *p* values adjusted for false discovery rate using Storey’s *q* value). Compounds with FC ≥ 1.40 or ≤ 0.71, *p* value < 0.05, and *q* value < 0.25 were deemed significantly altered. For multivariate statistics, features with near-constant values between the groups (the 30% features with the lowest relative standard deviation for all samples) were filtered out to reduce the complexity of the dataset, followed by auto-scaling. No other filtering, normalisation, transformation, or scaling methods were employed before statistical analysis. The data quality was assessed by the visual clustering of QC injections compared to the samples using principal component analysis (PCA) and hierarchical clustering (dendrogram), indicating the technical reproducibility.

## Results

### Reduced ANGPTL-3 and ANGPTL-4 levels in SOD1 ALS patients

We first assessed the ANGPTL-3/4/8 axis in four subgroups of human patients (Table 1), including healthy controls (*n* = 96), non-genetic or sporadic ALS (sALS) patients (*n* = 117), and ALS patients with mutations in the *SOD1* gene (*n* = 20) and the *FUS* (*n* = 14) or the *TARDBP* (encoding *TDP-43*) gene (*n* = 3).

**Fig. 1:**
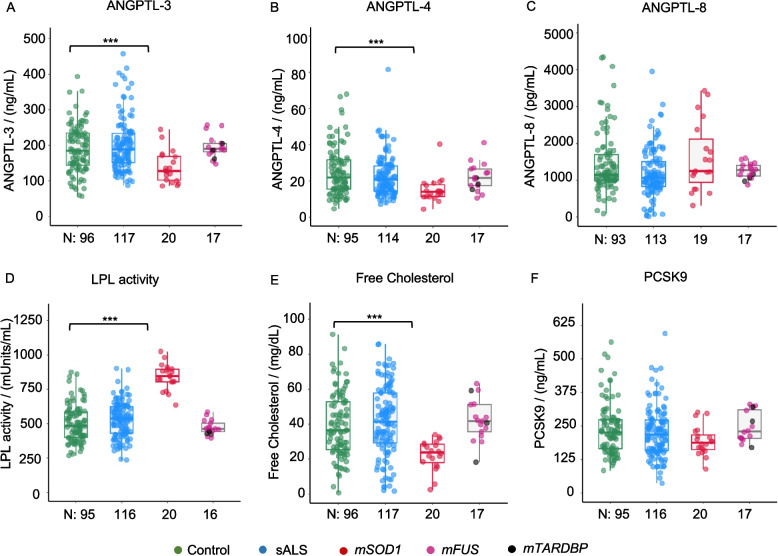
Reduced ANGPTL-3 and ANGPTL-4 in *SOD1* ALS patients. **A** and **B** show reduced serum ANGPTL-3 and −4 levels, respectively, as determined by ELISA in *SOD1* ALS patients, compared to healthy controls. **C** shows unchanged ANGPTL-8 in ALS patient groups. **D** represents increased LPL activity in *SOD1* ALS patients compared to healthy controls. **E** shows decreased free cholesterol in *SOD1* ALS patients compared to healthy controls. **F** represents unchanged serum PCSK9 levels in ALS patients, *SOD1* vs control (*p* = 0.08). *N* is the number of patients per group for each assay. Box plots show the median and interquartile range (IQR). Whiskers extend to the most extreme data point within 1.5 times the IQR. Individual data points are also shown. Statistical significance was determined by a two-way ANOVA, followed by Tukey’s HSD post hoc test for pairwise comparisons *p < 0.05, **p < 0.01, ***p < 0.001

*TARDBP* patients, available in small numbers due to the rarity of the mutation, were grouped with the FUS patients after verifying that the two groups were comparable in the readouts under study (the two subgroups are represented by different colours in the figures). None of the patients had received tofersen or any lipid-lowering drug at the time of sampling. Serum samples were obtained after overnight fasting. Serum ANGPTL-3 (Fig. 1A) and ANGPTL-4 levels (Fig. 1B) were significantly decreased (*p* < 0.001) in *SOD1* ALS patients compared to both healthy controls and non-genetic ALS patients (sALS). Alternatively, *FUS/TARDBP* patients were comparable to controls and non-genetic ALS. ANGPTL-8 levels (Fig. 1C) were similar across the four groups. The decreased serum levels of ANGPTL-3 and −4 were coherent with significantly higher activities of total serum LPL (Fig. 1D) in *SOD1* patients when compared to healthy controls or *FUS/TARDBP* subjects. We further explored if reduced ANGPTL-3 would correspond to a decrease in serum cholesterol.

Interestingly, total cholesterol (Additional file 2: Fig. S1) was reduced in *SOD1* patients but not in sporadic ALS or *FUS/TARDBP* patients compared to controls. De-esterified cholesterol (“free cholesterol”; Fig. 1E) was also significantly decreased in the *SOD1* patients (*p* < 0.001). Since patients with different genotypes displayed substantially different profiles in ANGPTL, LPL, and cholesterol levels, we also assessed the serum levels of proprotein convertase subtilisin/kexin type 9 (PCSK9), an enzyme that binds to LDL receptors to promote their lysosomal degradation [55]. Serum PCSK9 (Fig. 1F) was unchanged in non-genetic ALS and *FUS/TARDBP* patients compared to healthy controls. *SOD1* patients displayed non-significant (*p* = 0.08) levels of PCSK9 compared to controls.

We accounted for the effect of potential confounders, including sex, body mass index (BMI), and age by multivariate ANOVA. Age, sex, and BMI showed no significant interactions with the 4 subgroups (Additional file 1: Table S4), suggesting their effects are consistent across the population.

In summary, *SOD1* ALS patients, but not non-genetic patients, display a distinctive reduction in serum ANGPTL-3 and −4, associated with reduced total cholesterol.

### Serum lipidomics reveals dysregulation of lipid metabolism associated with ANGPTL

To characterise in detail the lipid phenotype associated with ANGPTL-3/4 levels in ALS patients, untargeted lipidomics at the molecular species level was performed on a subset of our cohort (Additional file 1: Table S5)—healthy controls (*n* = 40), non-genetic ALS patients (*n* = 37), and ALS patients with mutations in the *SOD1* gene (*n* = 20) and *FUS* (*n* = 14) or *TARDBP* genes (*n* = 3; FUS and TARDBP were pooled after demonstrating no difference). After lipid extraction and liquid chromatography (LC)-mass spectrometry (MS) analysis, we annotated 2344 compounds, including 971 based on *tandem*-MS spectral match (high confidence) and 1373 by accurate mass match (low confidence, tier 3) (Additional file 1). The annotations were validated with 74 lipid standards (see Methods). The peak intensities of annotated compounds were normalised by internal standards and used for uni- and multivariate statistics. The principal component analysis (PCA) scores plot with and without quality control (QC) samples (20 experimental replicates with injection duplicates of a pool of all samples) are shown in Additional file 2: Fig. S2A and B, respectively. The 20 QC technical replicates, each extracted with one sample batch, are tightly clustered, indicating suitable method reproducibility and appropriate performance for multivariate analysis. No unexpected trends, batch effects, or outliers were observed, showcasing good data quality.

**Fig. 2:**
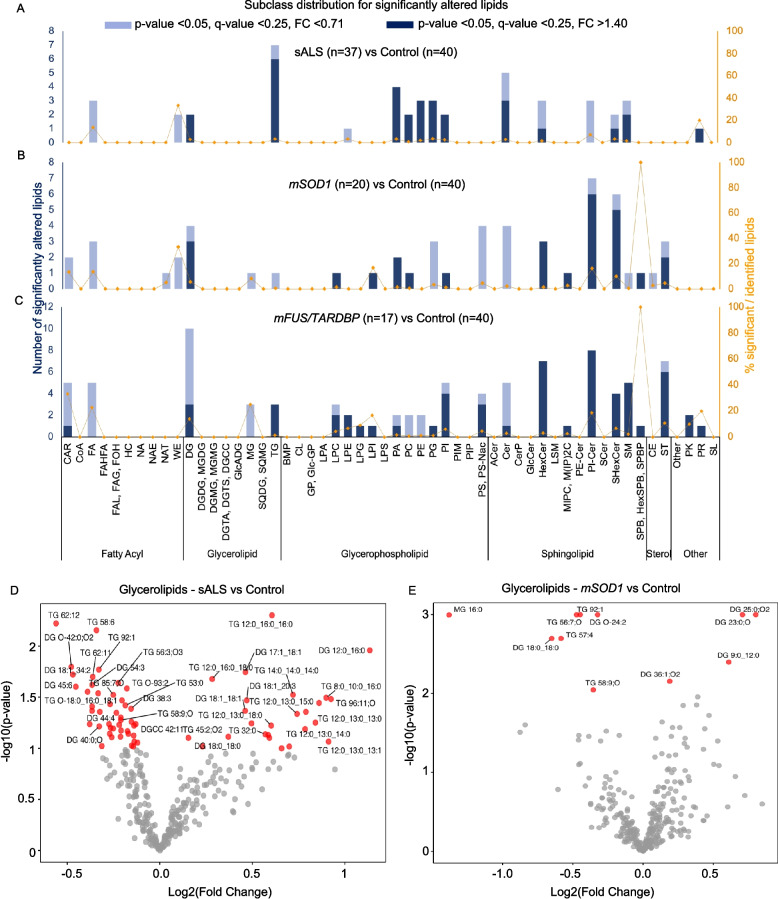
Serum lipidomics reveals dysregulation of lipid metabolism associated with ANGPTL. **A**–**C** show subclass distribution for significantly altered lipids revealed by untargeted global lipidomics. **A** Lipidome alterations in sALS patients compared to healthy controls. Increased TG for sALS patients. **B** Decreased TG and MG for *SOD1* patients compared to healthy controls. **C** Increased TG and decreased MG for *FUS/TARDBP* patients. **D**, **E** Volcano plots showing the fold change on the x-axis and FDR-adjusted *p* values on the y-axis for all lipid species belonging to the superclass glycerolipids (mainly composed of TG, DG, and MG) for sALS and *SOD1* patients, respectively, compared to healthy controls; TGs are decreased in *SOD1* patients while DGs mostly show an increase, while many TG species are increased in sALS patients

Compared to healthy controls, ALS subgroups displayed distinct lipidome alterations (Fig. 2A–C). Multiple (although not all) triglyceride (TG) and monoglyceride (MG) species were significantly decreased in patients with *SOD1* mutations (fold change < 0.71 and *p* < 0.05) (Fig. 2E), in agreement with their low ANGPTL-3/−4 phenotype. In contrast, we observed a significant increase in TG species in non-genetic (Fig. 2D) and patients with *FUS/TARDBP* mutations (Additional file 2: Fig. S2C). Several diglyceride species (DG; the first metabolite of TG upon LPL enzymatic activity) were increased in sporadic and genetic ALS. However, the DG/TG ratio rose only for *SOD1* subjects (Additional file 2: Fig. S2D), suggesting increased TG degradation and removal from circulation. For the *FUS* patients, the high TG and low DG levels resulted in low DG/TG ratio, suggesting reduced removal of TG from circulation.

Notably, several free fatty acid species (FA) were reduced in all ALS patient subgroups. Additionally, all ALS subgroups shared an elevation in one or more lipid subclasses enriched in myelin, such as hexosyl-ceramide (HexCer) and sphingomyelin (SM), as well as in lipids enriched in cell membranes (such as phosphatidic acid—PA and phosphatidylinositol—PI species), indicative of tissue and myelin damage. Notably, *SOD1* patients also displayed a decrease in several phosphatidylglycerol—PG and phosphatidylcholine—PC species; this pattern was not observed in sporadic or *FUS* patients.

As expected [56], genetic and sporadic ALS patients displayed a different age of onset (although a comparable ALSFRS at the sampling). In order to explore possible age impacts on the lipidome, we performed a multivariate analysis of variance (MANOVA) with the top 10 lipidomics intensities-derived principal components and clinico-demographical covariates. No significant effect of the age of sampling or onset, sex, BMI, or ALSFRS-R at sampling was detected (Additional file 1: Table S6).

Thus, the lipidomic analysis reveals distinct mutation-related profiles, with TG, MG, and DG alterations in *SOD1* patients compatible with their ANGPTL-3 phenotype.

### Serum ANGPTL-3 correlates with hypothalamic atrophy

**Fig. 3:**
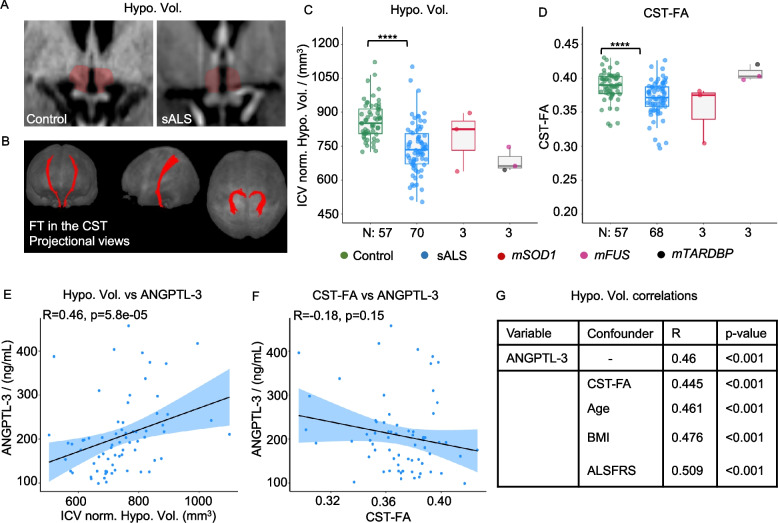
Serum ANGPTL-3 correlates with hypothalamic atrophy. **A** Representative 1.5-T MRI of healthy control with sALS patient showing reduced hypothalamic volume in sALS patients. **B** Fibre tracking method employed to detect fractional anisotropy values of the corticospinal tract in healthy controls and ALS patients. **C**. Reduced hypothalamic volume normalised with intracranial volume in all ALS patients compared to healthy controls, re-confirming hypothalamic atrophy in ALS. **D** Reduced fractional anisotropy of the corticospinal tract in ALS patients, exhibiting corticospinal tract atrophy in ALS. *N* is the number of patients per group. Box plots show the median and interquartile range (IQR). Whiskers extend to the most extreme data point within 1.5 times the IQR. Individual data points are also shown. Statistical significance was determined by a two-way ANOVA, followed by Tukey’s HSD post hoc test for pairwise comparisons *p < 0.05, **p < 0.01, ***p < 0.001. **E** Positive correlation of ICV normalised hypothalamic volume with serum ANGPTL-3 levels; Spearman’s rho of 0.46 with a *p* value of 5.8e − 05. **F** No correlation of CST-FA values with serum ANGPTL-3; Spearman’s rho of − 0.18 with a *p* value of 0.15. **G** Direct relationship between hypothalamic volume and serum ANGPTL-3 remains unchanged in the presence of confounders such as CST-FA, age, BMI, and ALSFRS as determined by multivariate correlation analysis. *R* is Spearman’s rho, and the *p* value is adjusted for the false discovery rate by the Benjamini–Hochberg method

Although at group level ANGPTL-3 levels were reduced compared to controls only in *SOD1*ALS patients, sporadic ALS patients displayed a substantial degree of variability. Since the metabolic phenotypes and the hypothalamic involvement (as revealed by the loss of hypothalamic volume—HV [40, 57]) appear only in a subset of ALS patients [40, 58, 59], we set out to explore how much of the ANGPTL-3 variability in the sporadic patients was correlated to the atrophy of the hypothalamus and/or of the motor system (assessed at the level of the corticospinal tract—CST). Thus, we quantified two imaging endophenotypes, i.e. HV corrected for intracranial volume and fractional anisotropy (FA) of the CST as fibre tracking measures of primary central motor system involvement (Fig. 3A, B) on T1w-MRI and DTI-MRI available for a subset of patients in our cohort (68 non-genetic ALS patients and 3 patients each with *SOD1* and *FUS* mutations). MRI data from healthy controls (*n* = 57, age and sex-matched) were used as a normal reference.

Although HV was on average lower in ALS patients than in controls, values for individual subjects displayed a substantial variability, from above the reference average to well below it (Fig. 3C). As anticipated, the FA of the CST was significantly reduced in ALS patients compared to healthy controls (Fig. 3D), and a very strong trend was observed for *SOD1* patients (*p* = 0.058). Importantly, in non-genetic ALS patients, serum ANGPTL-3 was directly correlated with HV (Fig. 3E, R = 0.46, *p* < 0.001) but showed no correlation with CST-FA (Fig. 3F, R = − 0.18, *p* > 0.05). Furthermore, in a multivariate analysis (Fig. 3G), the significant correlation of ANGPTL-3 with HV was not confounded by CST-FA, age, BMI, or disease severity (revised ALS functional rating scale (ALSFRS-R [60]). The limited size of the neuroimaging data for ALS patients with genetic mutations prevented further correlation analyses for those groups. On the other hand, ANGPTL-4 levels were not correlated with HV (or with CST-FA) upon univariate or multivariate analysis (Additional file 2: Fig. S3A–C). These data show that serum ANGPTL-3 alterations are related to the involvement of the hypothalamus in the disease process, suggesting that dysfunction of hypothalamic circuits may be involved in the decrease in systemic ANGPTL-3 and lipid dysregulation.

Reduced serum ANGPTL-3 and ANGPTL-4 in *SOD1* ALS murine models.

We then investigated if ALS murine models also display an alteration of the angiopoietin-like system, recapitulating the human phenotype. First, we elected to study the *SOD1*^*G93A*^ mouse since (i) the mutated gene makes it a relevant comparison to the *SOD1* ALS patients subgroup; (ii) it displays a predictable disease course, enabling the establishment of pre-symptomatic, early symptomatic, and fully symptomatic stages [61]; and (iii) it shows a metabolic phenotype in terms of weight loss and hypothalamic alterations [62, 63].

**Fig. 4:**
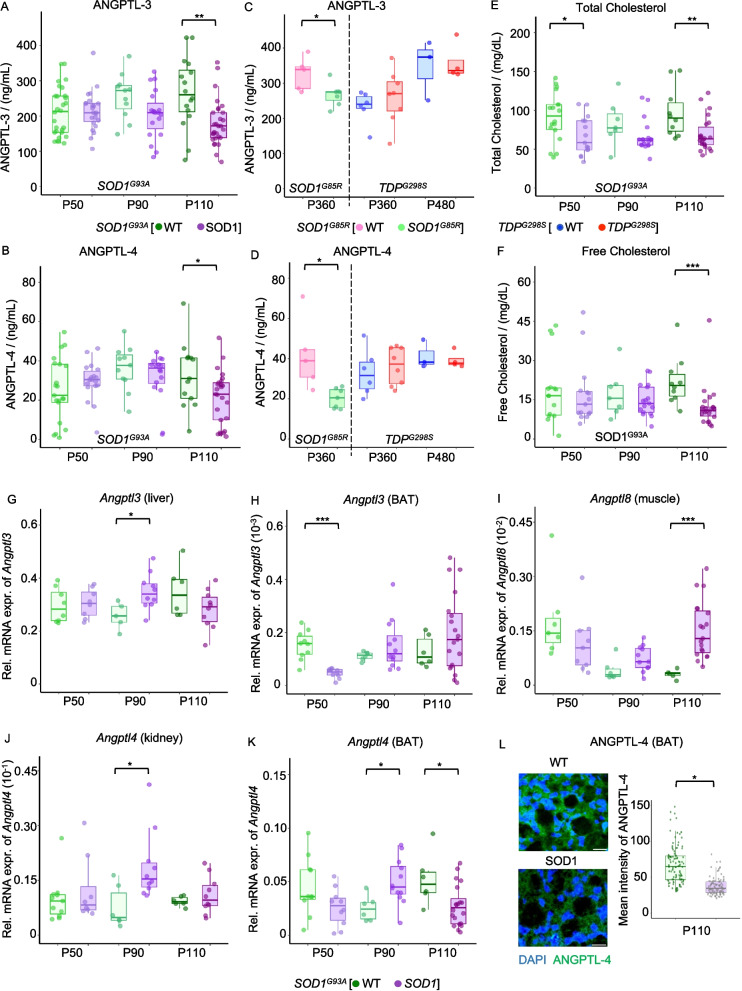
Reduced systemic ANGPTL-3, −4 and corresponding alterations in the periphery of *SOD1* ALS murine model. **A** and **B** show reduced circulating ANGPTL-3 and ANGPTL-4, respectively, in the fully symptomatic stage (P110) of *SOD1G93A* mice, compared to WT littermates of the same age. **C** and **D** show reduced ANGPTL-3 and −4 in fully symptomatic *SOD1G85R* mice (1-year-old) compared to WT; unchanged ANGPTL-3 and −4 in TDPG298S mice at any disease stage, respectively. **E** shows reduced total cholesterol in *SOD1G93A* mice across all disease stages compared to WT littermates. **F** shows reduced free cholesterol in the fully symptomatic stage. For *SOD1G93A* mice, P50 is the pre-symptomatic stage, P90 is the early symptomatic stage, and P110 is the fully symptomatic stage. **G** to **K** show relative mRNA expression of the corresponding *Angptl* gene with Gapdh, as measured via qPCR analyses in *SOD1G93A* mice compared with their corresponding wild-type littermates. **G** Increased *Angptl3* at P90 and a trend towards decreased *Angptl3* at P110 in mice liver. **H** Brown adipose tissue shows reduced *Angptl3* in *SOD1* mice at P50. **I** Increased *Angptl8* in the gastrocnemius muscle in *SOD1* mice at P110. **J** *Angptl4* is increased at P90 in the kidney of *SOD1* mice. **K** *Angptl4* is increased at P90, while reduced at P110 in the brown adipose tissue of *SOD1* mice. **L** Immunostaining of ANGPTL-4 in *SOD1* and WT mice at P110 shows reduced protein levels in *SOD1* mice, as determined by the mean fluorescent intensity. The scale bar is 20 µm. Box plots show the median and interquartile range (IQR). Whiskers extend to the most extreme data point within 1.5 times the IQR. Individual data points are also shown. Statistical significance was determined by a two-way ANOVA, followed by Tukey’s HSD post hoc test for pairwise comparisons *p < 0.05, **p < 0.01, ***p < 0.001. For *SOD1G93A* mice, P50 is the pre-symptomatic stage, P90 is the early symptomatic stage, and P110 is the fully symptomatic stage

We considered plasma samples obtained in male *SOD1*^*G93A*^ or male wild-type (WT) littermates at the age of P50 (50 days of age, pre-symptomatic), P90 (90 days of age, early symptomatic), and P110 (110 days of age, fully symptomatic). Notably, ANGPTL-3 (Fig. 4A) and −4 (Fig. 4B) levels were comparable in pre-symptomatic *SOD1* and WT littermates, showing a trend towards reduced levels at the early symptomatic stage (*p* = 0.06 for ANGPTL-3 at P90). At the fully symptomatic stage (P110), ANGPTL-3 (*p* < 0.05) and −4 (*p* < 0.01) levels were significantly reduced in *SOD1* mice compared to WT littermates.

We sought to verify if other ALS mouse models shared the ANGPTL phenotype observed in *SOD1* mice. Plasma samples from 1-year-old *SOD1*^*G85R*^ mice [64], a slow-progressing ALS mouse model, also showed reduced ANGPTL-3 (Fig. 4C) and ANGPTL-4 levels (Fig. 4D) compared to their WT littermates. Conversely, plasma samples from *TDP-43*^*G298S*^ mice (previously described [65, 66]) obtained at 12 and 16 months of age (corresponding to an early and fully symptomatic stage for this strain) showed no difference in plasma ANGPTL-3 (Fig. 4C) and ANGPTL-4 (Fig. 4D) compared to their same-aged littermates. We also looked at lipid correlates of ANGPTL, in particular, total and free plasma cholesterol levels in pre-symptomatic, early symptomatic, and fully symptomatic *SOD1* mice and their corresponding littermates (Fig. 4E and F). Surprisingly, total cholesterol (Fig. 4E) was reduced not only at the symptomatic stage, but also at the early symptomatic and pre-symptomatic stages. However, free cholesterol levels (Fig. 4F) were significantly reduced only at the symptomatic stage (as in humans). Furthermore, LPL activity in murine samples experienced a sudden increase at P110 (being lower than WT at P50 and P90; Additional file 2: Fig. S4K) in correspondence with the increase in ANGPTL-3; although absolute values cannot be compared across species, the overall trend in increase, upon decrease in ANGPTL-3 is consistent between humans and mice. These data show that the *SOD1* murine model largely recapitulates the ANGPTL system disruption and related cholesterol phenotype observed in human *SOD1* ALS patients.

Altered ANGPTL expression in peripheral organs of the *SOD1* ALS mouse model.

Next, we aimed to verify if systemic alterations in ANGPTL-3 and −4 observed in the *SOD1* mice were mirrored by alterations in expression at a specific organ/tissue level. We quantified the mRNA levels of *Angptl3,4* and *8* in the liver (the primary source of systemic ANGPTL-3), kidneys, skeletal muscle (gastrocnemius), and brown and white adipose tissues (BAT and WAT, respectively, primary sources of ANGPTL-4).

*Angptl3* was strongly expressed in the liver (Fig. 4G) but moderately expressed in kidney (Additional file 2: Fig. S3A), white (Additional file 2: Fig. S3C), and brown adipose tissues (Fig. 4H). It was marginally expressed in gastrocnemius (Additional file 2: Fig. S3B). Levels of *Angptl3* mRNA were comparable between *SOD1* and WT littermates at all time points and across tissues with two exceptions. In the liver, *Angptl3* showed an upregulation (*p* = 0.013) at the early symptomatic stage in *SOD1* mice, whereas a significant decrease (*p* < 0.001) was observed at the pre-symptomatic stage in BAT.

*Angptl4* mRNA was strongly expressed in BAT (Fig. 4K) and WAT (Additional file 2: Fig. S3F). It was moderately expressed in the kidney (Fig. 4J) and liver (Additional file 2: Fig. S3D) and less expressed in gastrocnemius (Additional file 2: Fig. S3E). Expression in the WAT and liver was unaffected across disease stages. For BAT, expression was substantially increased at the early symptomatic stage (P90, *p* = 0.018), followed by a significant decrease at the fully symptomatic stage (P110, *p* = 0.028). Interestingly, both kidney and gastrocnemius showed a significant upregulation of *Angptl4* only at the early symptomatic stage compared to same-aged WT littermates (*p* = 0.022 and *p* = 0.002 for kidney and gastrocnemius, respectively).

*Angptl8* was strongly expressed in the liver (Additional file 2: Fig. S3G) and moderately expressed in the kidney (Additional file 2: Fig. S3H), WAT (Additional file 2: Fig. S3I), BAT (Additional file 2: Fig. S3J), and gastrocnemius (Fig. 4I). Liver, WAT, and BAT levels of *Angptl8* mRNA were unchanged across disease stages. Interestingly, we found a strong upregulation of *Angptl8* in gastrocnemius in fully symptomatic animals (*p* < 0.001).

We further confirmed the downregulation of ANGPTL-4 at the fully symptomatic stage by immunofluorescent labelling in BAT samples from P110 *SOD1* mice (Fig. 4L). Immunoreactivity was detected in the cytoplasm of the adipose cells; *SOD1* samples displayed a substantially lower (*p* = 0.019) immunofluorescence intensity, confirming the decreased *Angplt4* mRNA expression.

In summary, the expression of *Angptl3,4* and *8* in multiple organs/tissues and across disease stages revealed the involvement of BAT in fully symptomatic (reduced *Angptl4*) stages; a transient disturbance in liver, kidney, and muscle at the early symptomatic stage; and the selective upregulation of *Angptl8* in muscle only in the fully symptomatic stage.

Altered triglyceride lipidome in symptomatic *SOD1* mice.

To characterise the lipid metabolism correlates of the changes in ANGPTL, we determined the plasma lipidome of *SOD1* mice at the pre-symptomatic, early symptomatic, and fully symptomatic stages by untargeted LC–MS/MS lipidomics. The LC–MS analysis of the mouse cohort resulted in the annotation of 1982 lipids, including 792 high-confidence *tandem*-MS spectral matches (obtained from operator-reviewed analytical annotation) and 1190 mass matches (low confidence). The annotations were validated with 57 lipid standards (see Methods). Relative standard deviations (RSDs) for internal standards added to each sample before lipid extraction varied between 3.6% and 16.8%, showing good reproducibility. The normalised peak intensities for annotated species (i.e. the peak intensity divided by the intensity of the most similar deuterated internal standard) were employed for uni- and multivariate statistical analysis (Additional file 3). The reproducibility of the 12 QC experimental replicates (with injection duplicates) was further assessed through multivariate analysis using PCA scores plots, where QC samples were tightly clustered (Additional file 2: Fig. S5A). No abnormal trends, outliers, or batch effects were noticed, indicating good data quality.

The partial least squares discriminant analysis (PLS-DA) analysis showed no clear separation between *SOD1* and WT samples at P50 (Additional file 2: Fig. S5B). However, the groups separated at P90 and P110, implying the appearance of relevant lipid metabolism alterations only at the symptomatic stage. In fact, at the pre-symptomatic stage (P50), no lipids were significantly altered between WT and *SOD1* mice after false discovery rate (FDR) adjustment.

**Fig. 5:**
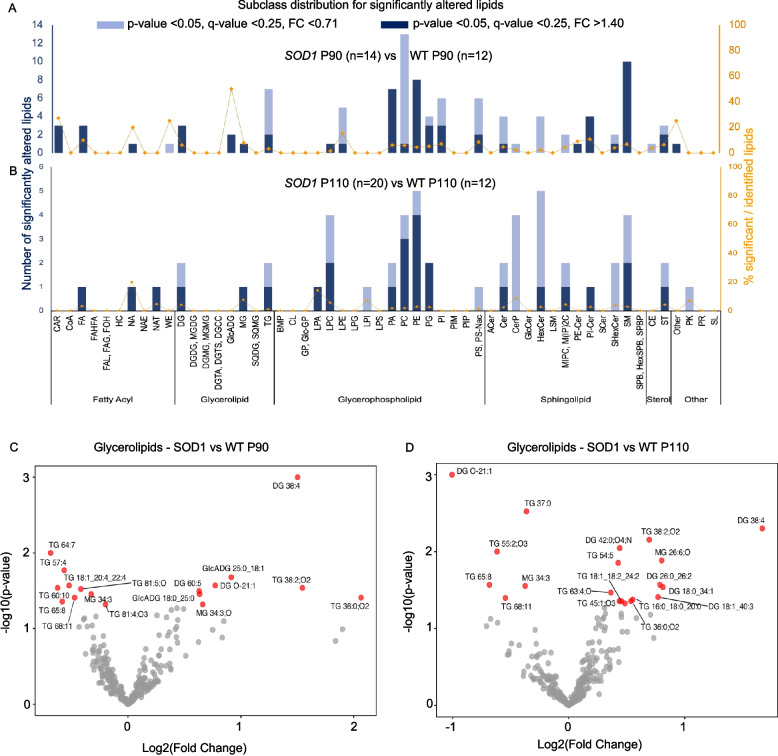
Altered triglycerides in the lipidome of symptomatic *SOD1* mice. **A** and **B** show subclass distribution for significantly altered lipids; decreased TG in *SOD1G93A* mice at the symptomatic stages, with increased MG and DG compared to WT littermates of the same age. **C**,** D** Volcano plots showing the fold change on the x-axis and FDR-adjusted *p* values on the y-axis for all lipid species belonging to the superclass glycerolipids (mainly composed of TG, DG, and MG) for *SOD1* mice compared to WT at P90 and P110, respectively

At the early symptomatic stage (Fig. 5A), several disruptions occurred in triglyceride species, with a decrease in most triglycerides and an increase in monoglyceride (MG), diacylglycerol (DG), and free fatty acid (FA) species, combined with a striking decrease in phosphatidylcholines (PC). At more advanced stages, a decrease in some TG species, but increase in some TGs and concomitant elevation of MG, DG, and FA species persisted. Moreover, almost all major lipid classes exhibited changes as the disease progressed. Sphingolipids such as SM, HexCer, and ceramides (Cer) showed a significant decrease in *SOD1* mice at P110 compared to WT (Fig. 5B). In agreement with the reduction of ANGPTL-3 at the symptomatic stage, the lipidome of glycerolipids revealed a significant decrease of TGs and increase of DG (Fig. 5C, D). Thus, the lipidome alterations observed in the *SOD1* mice recapitulate those observed in human *SOD1* patients and, with the progressive increase in the TG metabolites (DG, MG, FA) and decrease in TG and CE, are compatible with the reduction in ANGPTL-3.

Serum ANGPTL-3 directly correlates with hypothalamic AgRP/NPY and inversely with hypothalamic neuroinflammation in *SOD1* mice.

**Fig. 6:**
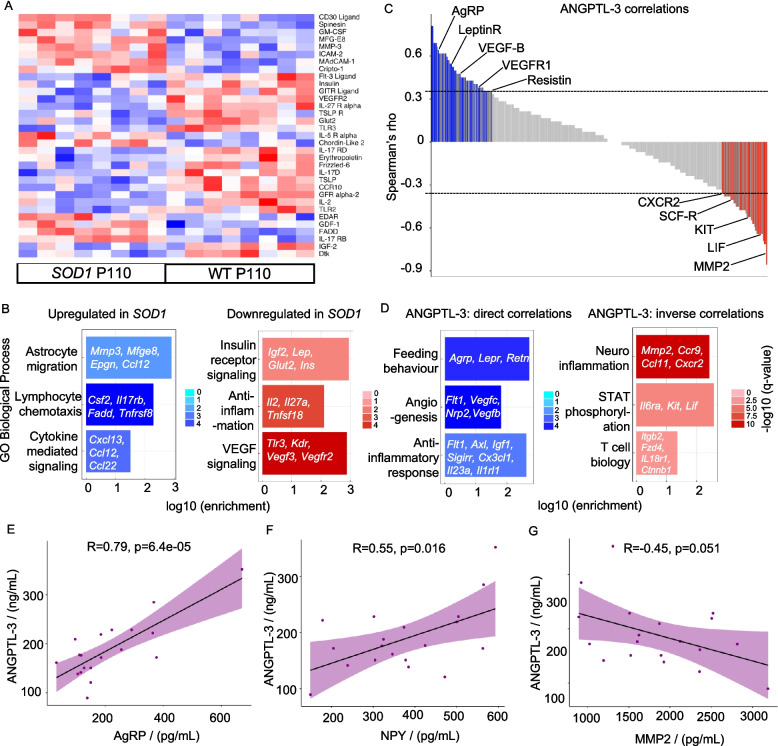
Peripheral ANGPTL-3 correlates with hypothalamic proteins in fully symptomatic *SOD1* mice. **A** Differentially expressed proteins in the hypothalamus of *SOD1G93A* mice at the fully symptomatic stage (P110) compared with same-age WT littermates, *N* = 8 each, as determined by an antibody array of 308 proteins. Several proteins involved in metabolism are reduced in the *SOD1* group, while pro-inflammatory proteins are increased. Bayesian statistics were used after normalising the data, and only the significant proteins obtained after a false discovery rate adjustment of < 0.05 were considered differentially expressed. **B** shows the gene ontology for proteins that are upregulated and downregulated in *SOD1* mice. **C** shows the correlation of systemic ANGPTL-3 levels of *SOD1* mice with the proteins in the antibody array. All proteins correlating directly or inversely with an estimate of ± 0.35 are marked in dark grey. In contrast, statistically significant ones are marked blue or red depending on the direct or inverse correlation estimates. **D** represents the selected gene ontology enrichment analysis of the proteins directly and inversely correlated with systemic ANGPTL-3, respectively. The enrichment for **B** and **D** was done via Stringdb against the gene set of *Mus musculus*, with enriched genes adjusted for false discovery rate. **E** and **F** show a positive correlation of systemic ANGPTL-3 with hypothalamic AgRP (*R* = 0.79, *p* = 6.4e − 05) and NPY (*R* = 0.55, *p* = 0.016), respectively, as measured by ELISA in a larger cohort (*n* = 19) of *SOD1* P110 mice. **G** shows an inverse correlation of hypothalamic MMP2 with systemic ANGPTL-3 (*R* = − 0.45, *p* = 0.051) in *n* = 19 *SOD1* P110 mice, thereby validating the results from the antibody array. For **E** to **G**, *R* is the Pearson’s correlation coefficient

In ALS patients, serum ANGPTL-3 levels directly correlated with the appearance of hypothalamic atrophy, suggesting that dysfunction of hypothalamic circuits may be responsible for the decrease in systemic ANGPTL-3 and lipid dysregulation. To investigate this connection at the molecular and circuit level, we explored the connections between hypothalamic phenotypes and ANGPTL phenotypes in the *SOD1* animals. We first contrasted the proteomic profile of the hypothalamus using a semi-quantitative 308 targets antibody from *SOD1* (*n* = 8) and WT (*n* = 8) mice at the fully symptomatic stage, when the reduction of ANGPTL-3 in *SOD1* mice was detected. Nineteen proteins (including FLT3 ligand, insulin, GITR ligand, GLUT2, and erythropoietin) were decreased and 14 proteins (amongst them MMP3, ICAM2, IL5Ra, CD30 ligand) were increased in *SOD1* mice (Fig. 6A; the complete list of differentially expressed proteins is reported in Additional file 4). The gene ontology (GO) analysis of the dysregulated proteins underscored the involvement of pathways involved in metabolism (insulin receptor signalling, regulation of cholesterol transport), vascular permeability (VEGF signalling pathway, regulation of angiogenesis), and neuroinflammation (cytokine-mediated signalling, leukocyte proliferation and migration) in the ALS hypothalami (Fig. 6B).

Next, we performed a linear correlation between the hypothalamic level of each of the 308 proteins with the levels of plasma ANGPTL-3. After correction for multiple testing, 34 proteins were significantly correlated with peripheral ANGPTL-3 with an *R* value of above 0.35 and 26 proteins were significantly inversely correlated with an *R* value of below − 0.35 (Fig. 6C; full list in Additional file 4). The GO analysis of correlated or anti-correlated proteins revealed a direct correlation between terms involved in feeding behaviour (agouti-related peptide (AgRP), leptin receptor, and resistin) and peripheral ANGPTL-3 (i.e. higher hypothalamic levels of AgRP corresponded to preserved ANGPTL-3 levels; Fig. 3C). Likewise, proteins involved in maintaining vascular permeability (CX3CL1, ITGAM, ICAM1, SELE, SELL, FLT1, NRP2, VEGFC, and VEGFB) and, notably, anti-inflammatory proteins (FLT1, AXL, IGF1, and SIGIRR) were also directly correlated with ANGPTL-3 levels. Conversely, the levels of pro-inflammatory mediators (MMP2, CCR3, CCR9, CCR10, PPBP, CCL11. and CCR2), members involved in T-cell biology (IL1R1, FZD4, CTNNB1, and ITGB2), and activators of NF-kB (TLR2, TNFSF4, KIT, and IL18R1) and STAT3 (IL6RA, KIT, and LIF) were elevated in mice with reduced ANGPTL-3 levels (Fig. 6D).

We sought to validate the array data with an independent platform (ELISA) and a larger mouse cohort (*n* = 19) considering two neuropeptides (AgRP and NPY) that are easily quantified by ELISA and known to be highly involved in feeding behaviour and energy balance: in fully symptomatic *SOD1* mice, we once again detected the positive correlation between hypothalamic AgRP (Fig. 6E), and neuropeptide Y (Fig. 6F) and plasma ANGPTL-3. We also opted to reconfirm one neuroinflammation marker, detecting the inverse relationship of ANGPTL-3 and MMP-2 (*n* = 19; Fig. 6G) in the ELISA dataset. A similar analysis for ANGPTL-4 revealed 27 proteins directly and 14 proteins inversely significantly correlated (Additional file 2: Fig. S6A) with a similar trend identified for ANGPTL-3 (pro-inflammatory proteins inversely correlated with ANGPTL-4, anti-inflammatory proteins directly correlated with ANGPTL-4; Additional file 2: Fig. S6B). Reverification by ELISA showed similar trends but with smaller *R* and borderline significance between ANGPTL-4 and AgRP and NPY on one side and between ANGPTL-4 and MMP2 on the other side (Additional file 2: Fig. S6C–E). When combined, these data identify that alterations of the AgRP/NPY hypothalamic circuit are highly related to ANGPTL-3/4 in the periphery and demonstrate that upregulation of neuroinflammatory responses in the hypothalamus correlates with the disturbance of systemic ANGPTL-3 and the ensuing lipid dyshomeostasis.

## Discussion

**Fig. 7:**
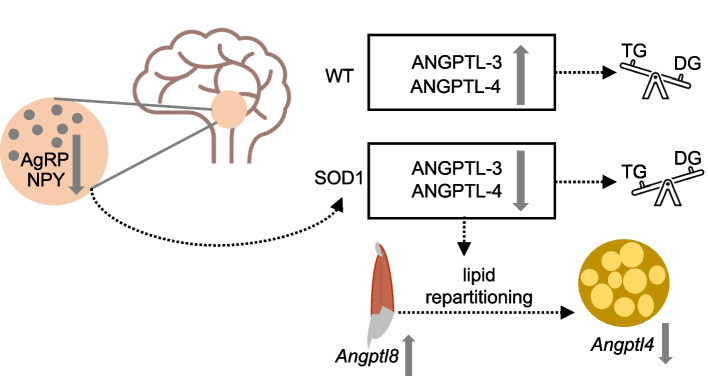
Interpretative framework. Hypothalamic dysfunction including neuroinflammation (not shown) and alterations in the AgRP-NPY circuitry are associated with a systemic decrease in ANGPTL-3 and ANGPTL-4, which modifies the uptake of triglycerides, and depending on the relative tissue expression of Angptl3-4–8, lipids are partitioned away from the muscle and towards the brown adipose tissue

We presented converging evidence from human patients and murine models of an alteration of the ANGPTL system in ALS related to hypothalamic dysfunction and directly correlated with lipid abnormalities (summarised in Fig. 7). Notably, the ANGPTL phenotype is prominent in *SOD1* genetic ALS patients (and murine models) but not in *FUS/TARDBP*. The ANGPTL alteration constitutes one of the first molecularly defined mechanisms for altered systemic lipid metabolism in ALS, connecting it to the dysfunctions of hypothalamic circuits.

We found a downregulation of systemic ANGPTL-3 and −4 levels in *SOD1* patients, corresponding to the upregulation of LPL activity and the increase in the DG-MG/TG ratio (indicating degradation of TG and upregulation of their break-down products), in agreement with the physiological role of the ANGPTL-3–4–8 axis [67–70]. The pattern of ANGPTL-3 and lipid profiles in ALS patients is consistent with the clinical experience on ANGPTL-3: a decrease in ANGPTL-3 and −4 leads to a reduction in TG and cholesterol [22, 27]. This phenotype is recapitulated in the *SOD1* mouse models: systemic ANGPTL-3, −4 and free cholesterol levels are reduced in the fully symptomatic stage (P110) both in fast-progressing *SOD1*^*G93A*^ mice and in slow-progressing *SOD1*^*G85R*^, but not in TDP^G298S^ mice, in agreement with the human subjects. Recently, ANGPTL-3 was identified as a component of HDL that regulates cholesterol efflux [71]. Since the *SOD1* model is validated by the human data, we have exploited it to identify the mechanisms and the physiological implications of the systemic changes in ANGPTLs. At the symptomatic stage, a strong trend towards decreased *Angptl3* mRNA in the liver (the most crucial source of ANGPTL-3) of *SOD1* mice was detected, which may account for the reduced serum levels. Likewise, *Angptl4* was decreased significantly in the BAT (a major source of systemic ANGPTL-4 [72]) of symptomatic *SOD1* mice, most likely accounting for the decrease in serum ANGPTL-4. Strikingly, *Angptl8* was strongly upregulated in the muscles of fully symptomatic *SOD1* mice, concurrent with the downregulation of BAT *Angptl4*. This pattern is coherent with the role of the ANGPTL system in regulating lipid partitioning between storage and oxidative tissues [39]. In normal conditions, feeding upregulates ANGPTL-3/ANGPTL-8 complexes in muscles and other organs, shifting lipid uptake from muscle to adipose tissue by decreasing LPL activity in the former and increasing it in the latter (by competing with ANGPTL-4-dependent inhibition [73]). However, fasting induces ANGPTL-4 in the adipose tissue [35], which reduces lipid extraction in adipose tissue promoting the partitioning of lipids towards oxidative tissues. In the *SOD1* animals, the simultaneous elevation of muscle *Angptl8*, with no change in *Angptl3*, and downregulation of *Angptl4* in BAT points towards the repartitioning of lipids away from muscles and towards the BAT, strikingly resembling the pattern observed in metabolic adaptation related to thermogenesis [74–76]. These findings are in overall agreement with the disturbances detected in circadian rhythm and related phenomena [77, 78] implying a sustained thermogenesis even during the sleep phase.

The ANGPTL axis alterations are reflected in the serum lipidome of genetic ALS patients. TGs are solely decreased in *SOD1* patients, whereas in the *FUS/TARDBP* group, we found an increased retention of TGs in the blood. The ratio of DGs to TGs is increased only in the *SOD1* group, which agrees with increased LPL activity and reduced ANGPTL-3 levels. The mouse lipidome is in agreement with the human findings: *SOD1* mice at P90 (early symptomatic) and P110 (fully symptomatic) display reductions in several TGs accompanied by an increase in catabolic products (DGs and MGs), and in agreement with increased clearance of triglycerides previously reported in *SOD1* mice [79].

Our TG data are mostly in agreement with previous lipidome reports: some studies [80, 81] found reduced MGs but not TGs alterations, although in our cohort this phenotype is restricted to genetic patients. Other human lipidomic studies reported increased DGs but not TGs compared to controls. DGs and TGs increased in ALS patients after 12 months [82] or TGs only [83], though the latter found decreased TGs in patients with faster progression rate compared to normal progressors. Another study [84] found increased or decreased TGs subtypes, depending on their chemical structure, in the serum of ALS patients.

Notably, possible divergences between our findings and other studies may be due to the resolution of the lipidome. Other researchers have opted to extract lipids according to the Bligh-Dyer protocol [80, 82], which provides extraction efficiencies comparable to our chosen Folch method. A combination of metabolomics and lipidomics has been employed [84] to achieve relative quantifications of lipids, whereas we focussed on an in-depth global lipidomics approach. Further, MS/MS fragmentation patterns for identification of lipids at the class level [83] were extended to lipid identification at the molecular species level in our study.

Since the human and mouse lipidomes were not analysed simultaneously, only their general trends can be compared. In this respect, TG and DG patterns show substantial similarities between human and mouse samples: in both cases, DGs are increased; TGs are reduced both in patients and in the early symptomatic mice and largely decreased (although not all) also in fully symptomatic mice. Likewise, sphingolipids are elevated in patients as well as in early symptomatic mice. Some divergences do appear when patients are compared to fully symptomatic mice: at this stage, food intake for mice may be less controlled than for humans.

More substantial divergences are observed in the levels of the downstream degradation products of DG: MG and FFA are elevated in mice but reduced in patients. This pattern may be related to the baseline higher concentration of FFA in mice than in humans [85, 86], possibly related to a physiological difference that requires higher levels of FFA in mice to sustain their higher metabolic rate [87].

Although our findings point in the direction of an ANGPTL-3/4 alteration contributing to lipid metabolism abnormalities, they do not imply this is the only existing alteration; additional events, e.g. regulated through other hypothalamus-controlled physiological loops, may contribute to the overall phenotype.

Furthermore, our human data agrees with the mouse lipidomes, showing an increase in several species of sphingomyelins in *SOD1* mice but only at symptomatic stages (P90 and P110). These findings are compatible with the hypothesis that serum sphingomyelins are markers of tissue damage, specifically myelin and neuron damage [88–90]. The elevation in sphingomyelins has also been reported by previous lipidome studies in humans [83, 84, 91–93] as well as in mice [94–96]. However, whether these changes were intrinsic to the disease or a manifestation of accumulating degeneration remained unsettled. Our study points to the relationship between an increase in sphingomyelins as the disease progresses, followed by a decline at the end of the clinical course when extensive degeneration has already occurred. Interestingly, the sphingomyelin elevation occurs predominantly in *FUS* and *SOD1* patients but much less in sporadic ALS; this heterogeneity may point to a more severe lower motor neuron damage in genetic patients or to a more diverse case mix included in the sporadic cohort. This distinction could also reflect mutation-specific disruptions in lipid metabolism that diverge from mechanisms driving sporadic ALS.

Several mechanisms have been proposed to explain the lipid metabolism alterations in ALS [4]. Systemic mitochondrial dysfunction has been hypothesised: ultrastructural changes in mitochondrial cristae and intramitochondrial protein aggregations were identified by electron microscopy in postmortem brain samples from ALS patients [97]. In particular, the *C9ORF72* gene—responsible for another common genetic mutation in ALS patients—was suggested to be involved in the stabilisation of the inner membrane complex, and its mutation would thereby impair mitochondrial complex I formation [98]. Alternatively, blood epigenomic analysis has supported a disturbance in systemic lipid biosynthesis and transport [99]. Third, abnormal muscle metabolism has been implicated in lipid disturbances since *TDP43*-overexpressing mice display reduced glucose import and insulin resistance [100, 101]. Perturbations in muscle cholesterol transport and cholesterol overload correlating to disease severity have been reported [102].

We found that hypothalamic pathology strongly correlates with ANGPTL phenotype in human patients and mice models. Our human neuroimaging data display hypothalamic atrophy in ALS, in agreement with previous reports [40, 63, 103]. Furthermore, hypothalamic atrophy, but not corticospinal involvement, correlates with ANGPTL-3, implying that the reduced ANGPTL-3 might be a consequence of hypothalamic involvement (also observed in human pathology studies [104, 105]). Although the correlation between ANGPTL-3 and HV appears robust (and not affected by confounders), it is likely that factors other than HV, such as nutritional status, clinical phenotype, and genetic heterogeneity, may contribute to determine the overall ANGPTL-3 spectrum of values. Of note, our data suggest that ANGPTL-3 levels might be used as a potential biomarker of hypothalamic involvement for stratification or disease monitoring purposes.

The whole-hypothalamus proteomic data point to at least three main pathophysiological processes unfolding in the hypothalamus, which are directly or inversely related to ANGPTL-3/4 lipid abnormalities.

First, increased neuroinflammation mediators and decreased anti-inflammatory proteins are correlated to pathologically low ANGPTL-3/4 levels. These findings are in agreement with the appearance of neuroinflammatory changes in disease-affected areas in ALS, both in the spinal cord [61] and in the brain [106], and support the direct involvement of the hypothalamus.

Second, multiple mediators of vascular permeability and angiogenesis in the hypothalamus correlate to systemic ANGPTL-3/4. Indeed, dysfunction of the blood–brain barrier (BBB) integrity in ALS has been reported both in disease-affected areas from pathology specimens [107] as well as in mouse models [108–110] and its detection in the hypothalamus is an additional indication of the involvement of this structure in the disease processes. Intriguingly, the integrity of the BBB is regulated by MCH neurons, whose activity enables the controlled access of leptin to medial-eminence neurons [111]. Since MCH neurons are dysfunctional in ALS [104, 112], the correlation between BBB alterations and peripheral ANGPTL-3 levels may be mediated by the dysfunction of the melanocortinergic circuit.

Third, a low systemic ANGPTL-3 correlated to lower levels of hypothalamic AgRP and neuropeptide Y (NPY) (confirmed by two independent assays); these data suggest a link between the peripheral ANGPTL-3 lipid phenotype and a neurochemically identified hypothalamic circuit. Our findings are consistent with the known role of the AgRP/NPY system in stimulating food intake through the inhibition of the parabrachial neurons [113] and their role in shifting the metabolism towards lipid storage [114]; furthermore, AgRP deficiency suppresses serotonergic signalling in the dorsal raphe nucleus leading to increased thermogenesis [115]. In our data, higher hypothalamic levels of AgRP correlated with higher ANGPTL-3 (and therefore reduced lipid utilisation), matching the anticipated shift to lower lipid metabolism and reduced need for thermogenesis. Interestingly, AgRP and NPY neurons project to MCH neurons and together regulate thermogenesis [115]. It is also plausible that this correlation between AgRP/NPY and ANGPTL-3 is indirectly caused by an altered food and energy intake behaviour observed in ALS. Thus, interventions at the level of AgRP/NPY circuits may contribute to normalising the systemic metabolism in ALS.

The present study displays some limitations which are difficult to avoid. The nutritional and circadian state of mice and ALS patients are not identical due to their different active phase circadian rhythms and dietary habits. Since patients did not follow a specific dietary schedule before serum sampling, mice were also fed ad libitum to mimic the unrestricted feeding patterns of humans. Furthermore, ANGPTL-3 does not regulate cholesterol metabolism in mice to the same extent it does in humans: in fact, ANGPTL-3 deficiency in humans markedly reduces VLDL-ApoB production (impacting LDL-C precursors) and reduces cholesterol and triglycerides, while in mice, it primarily accelerates VLDL-TG clearance, mainly affecting triglyceride levels [116, 117]. Thus, the relationship between cholesterol levels and ANGPTL-3 may be mechanistically different in the two species; it is worth noting that, conversely, the decrease in esterified cholesterol points to an increased activity of LPL in both species as a consequence of ANGPTL-3 downregulation.

A direct comparison between TARDBP and FUS patients was not possible because of the reduced number of TARDBP patients, due to the rarity of this genotype. Although plasma samples would constitute the golden standard for lipidomics [118], plasma is not routinely stocked in biobanks and was not available for human patients; of note, murine and human samples were not compared to each other (no serum vs plasma comparison was performed) but to their internal references (control subjects or WT animals). Neuroimaging data about the correlation of hypothalamic volume with serum ANGPTL-3/4 are available in sufficient numbers only for sporadic ALS patients; the relative rarity of genetic ALS patients resulted in the unavailability of matched clinical and neuroimaging datasets for most of them.

## Conclusions

This study provides evidence from human patients and mouse models for hypothalamic-driven lipid metabolism alterations in ALS, mediated by ANGPTL-axis derangement and possibly aimed at sustaining a deregulated thermogenesis in adipose tissue. Thus, serum ANGPTL-3 levels may potentially constitute a new stratification parameter and a biomarker of response for metabolic interventions in ALS.

## Supplementary Information

Additional file 1. Tables S1–S6. Table S1 Clinico-demographic characteristics of the neuroimaging cohort. Table S2 List of primers used for qPCR. Table S3 List of antibodies used for immunofluorescence staining. Table S4 MANOVA results for ANGPTLs. Table S5 Clinico-demographic characteristics of the lipidomics cohort. Table S6 MANOVA results for lipidomics.

Additional file 2. Figures S1–S6. Fig. S1 Reduced total cholesterol in mSOD1 patients. Fig. S2 Lipidome of ALS patients separate based on genetics. Fig. S3 Lack of association between serum ANGPTL4 and neuroimaging endophenotypes. Fig. S4 Altered expression levels of ANGPTLs in peripheral tissues. Fig. S5 The lipidome of SOD1 animals is different from their WT counterparts at the symptomatic stages. Fig. S6 Peripheral ANGPTL-4 correlates with hypothalamic AgRP.

Additional file 3. An excel sheet with the normalised lipidomics dataset of the human and mouse samples.

Additional file 4. An excel sheet with the normalised intensity values of proteins from the antibody array, along with the differentially expressed and correlated proteins.

## Data Availability

All data generated or analysed during this study are included in the article and the additional information files.

## Abbreviations

AgRP: Agouti-related protein
ALS: Amyotrophic lateral sclerosis
ALSFRS-R: Amyotrophic lateral sclerosis functional rating scale—revised
ANGPTL: Angiopoietin-like protein
ASO: Antisense oligonucleotide
BAT: Brown adipose tissue
BBB: Blood-brain barrier
CE: Cholesterol ester
CST-FA: Corticospinal tract—fractional anisotropy
DG: Diglyceride
DTI: Diffusion tensor imaging
FA: Fatty acyl
FDR: False discovery rate
HDL: High-density lipoprotein
HexCer: Hexosyl-ceramide
HV: Hypothalamic volume
ICV: Intracranial volume
LDL: Low-density lipoprotein
LPL: Lipoprotein lipase
MG: Monoglyceride
MRI: Magnetic resonance imaging
MRI: Magnetic resonance imaging
NPY: Neuropeptide Y
PCA: Principal component analysis
PCSK9: Proprotein convertase subtilisin/kexin type 9
SM: Sphingomyelin
SOD1: Superoxide dismutase 1
TC: Total cholesterol
TFAS: Tract-wise fractional anisotropy statistics
TG: Triglyceride
TIFT: Tensor Imaging and Fibre Tracking
WAT: White adipose tissue
WT: Wild type
