# Supplementary material for: Disruption of the angiopoietin-like system connects lipid homeostasis and hypothalamic dysfunction in ALS
Source: BMC Med. 2026 Mar 3;24:210. doi: 10.1186/s12916-026-04749-4 (PMC13064175; doi:10.1186/s12916-026-04749-4)
Supplement: Supplementary file 1 — Additional file 1. Tables S1–S6. Table S1 Clinico-demographic characteristics of the neuroimaging cohort. Table S2 List of primers used for qPCR. Table S3 List of antibodies used for immunofluorescence staining. Table S4 MANOVA results for ANGPTLs. Table S5 Clinico-demographic characteristics of the lipidomics cohort. Table S6 MANOVA results for lipidomics. [file 12916_2026_4749_MOESM1_ESM.docx]

**Table S1 Clinico-demographic characteristics of the neuroimaging cohort**

| Supp.Table 1 | Healthy Control | sALS | *mSOD1* | *mFUS* | *mTARDBP* | p-value^2^ |
| --- | --- | --- | --- | --- | --- | --- |
| Number of patients ^1^ | 57 | 70 | 3 | 2 | 1 | - |
| Female  Male | 28 (49%)  29 (51%) | 36(51%)  34(49%) | 2(67%)  1(33%) | 1(50%)  1(50%) | 0  1(100%) | >0.9 |
| Age at sampling | 69(51,74) | 67(57,72) | 60(51,66) | 52 (49, 54) | 50 (50, 50) | 0.4 |
| Age at onset | - | 66(60,71) (n=54) | 58(50,64) (n=3) | 50(49,51) (n=2) | 49(49,49) (n=1) | 0.059 |
| ALSFRS-R  at sampling | - | 42(35,44) (n=60) | 44(41.5,44.5) (n=3) | 44.5 (44.0, 45.0) (n=2) | 39.0 (39.0, 39.0) (n=1) | 0.4 |
| BMI at sampling | - | 25.6(22.4,28.6) (n=59) | 25.7(25.5,27.8) (n=3) | 25.5 (25.4, 25.7) (n=2) | 29.8 (29.8, 29.8) (n=1) | 0.7 |
| Disease duration (months) | - | 12(7,18)  (n=64) | 24(22,28)  (n=3) | 10 (5, 16) (n=2) | 21 (21, 21) (n=1) | 0.13 |

^1^ n (%), median (IQR) ^2^ Kruskal-Wallis rank sum test

Data are median (quartiles) for skewed data. ALSFRS-R, Amyotrophic lateral sclerosis functional rating scale – revised; BMI, Body Mass Index; Disease duration is the number of months from the date of onset until the date of sampling.

**Table S2 List of primers used for qPCR**

| **Gene** | **Sequence** |
| --- | --- |
| *Gapdh* | forward: 5' -TGGATCTGACTGCCGC- 3'  reverse: 5' -TGCCTGCTTCACCTTC- 3' |
| *Angptl3* | forward: 5' -GAGCACCAAGAACTACTCCCC- 3' reverse: 5' -ATAAACGGCAGAGCAGTCGG- 3' |
| *Angptl4* | forward: 5' -CTTCCACTCTATCCCACGGC- 3' reverse: 5' -TAGCGGCCCTTCCATGTTTT- 3' |
| *Angptl8* | forward: 5' -CAGAGCCACCTCTTATGGGC- 3' reverse: 5' -GCATCCAGGTAGTCTCAGGC- 3' |

**Table S3 List of antibodies used for immunofluorescence staining**

| **Antibody** | **Host** | **Company** | **Catalog. nr.** | **Dilution** |
| --- | --- | --- | --- | --- |
| Angiopoietin like 4 | Rabbit | Abcam | ab196746 | 1:200 |
| DAPI 405 |  | Thermo Fisher | 62247 | 1:1000 |
| Anti-Rabbit AF 488 | Donkey | Invitrogen | A21206 | 1:500 |

**Table S4 Multivariate Analysis of Variance (MANOVA) Results for ANGPTLs**

| Supp.Table 4 | Df | Pillai | Approx F | Num Df | Den Df | Pr(>F) |
| --- | --- | --- | --- | --- | --- | --- |
| Group:Sex | 3 | 0.033094 | 0.8440 | 9 | 681 | 0.5756450 |
| Group:Age at sampling | 3 | 0.052912 | 1.3585 | 9 | 681 | 0.2033362 |
| Group:BMI at sampling | 2 | 0.055515 | 1.0944 | 6 | 230 | 0.36645 |
| Group: ALSFRSR at sampling | 2 | 0.017103 | 0.3048 | 6 | 212 | 0.93399 |
| Group: Age at onset | 2 | 0.011603 | 0.2062 | 6 | 212 | 0.97460 |

This table presents the results of a two-way Multivariate Analysis of Variance (MANOVA) conducted to assess the combined effects of subgroups (Controls, sALS, mSOD1 and mFUS/TARDBP) and clinico-demographic characteristics (Sex, Age and BMI) on the levels of ANGPTL-3,4&8. The significance of the multivariate effects was assessed using Pillai's Trace.

**Table S5 Clinico-demographic characteristics of the lipidomics cohort**

| Supp.Table 5 | Healthy Control | sALS | *mSOD1* | *mFUS* | *mTARDBP* | p-value^2^ |
| --- | --- | --- | --- | --- | --- | --- |
| Number of patients ^1^ | 40 | 37 | 20 | 14 | 3 | - |
| Female  Male | 28 (49%)  29 (51%) | 36(51%)  34(49%) | 12(60%)  8(40%) | 6(43%)  8(57%) | 0  3(100%) | 0.5 |
| Age at sampling | 60 (48, 70) | 65 (60, 71) | 51 (45, 62) | \| 47(41,56) \|  \| \| --- \| --- \| | 57 (51, 72) | 0.002 |
| Age at onset | - | 63 (57,70) (n=35) | 50 (42,61) (n=20) | 46 (33,53) (n=13) | 56 (49,71) (n=3) | 0.059 |
| ALSFRS-R  at sampling | - | 44 (40, 45) (n=33) | 42 (35, 44) (n=19) | 41 (37, 44) (n=13) | 39 (37, 39) (n=3) | 0.2 |
| BMI at sampling | - | 24.8 (22.9, 26.8) (n=33) | 25.7 (22.4, 27.6) (n=20) | 25.5 (22.9, 26.5) (n=12) | 25.7 (24.4, 29.8) (n=3) | 0.7 |
| Disease duration (months) | - | 27 (20, 41) (n=25) | 22(12,38) (n=20) | 12 (9, 18) (n=12) | 10 (9, 21)  (n=3) (n=2) | 0.3 |

^1^ n (%), median (IQR) ^2^ Kruskal-Wallis rank sum test

Data are median (quartiles) for skewed data. ALSFRS-R, Amyotrophic lateral sclerosis functional rating scale – revised; BMI, Body Mass Index; Disease duration is the number of months from the date of onset until the date of sampling.

**Table S6 MANOVA Results for lipidomics**

| Supp.Table 6 | Df | Pillai | Approx F | Num Df | Den Df | Pr(>F) |
| --- | --- | --- | --- | --- | --- | --- |
| Group:Sex | 3 | 0.28886 | 0.9056 | 30 | 255 | 0.61230 |
| Group:Age at sampling | 3 | 0.37879 | 1.2283 | 30 | 255 | 0.19942 |
| Group:BMI at sampling | 2 | 0.19761 | 0.9210 | 20 | 168 | 0.56133 |
| Group: ALSFRSR at sampling | 2 | 0.19723 | 0.9190 | 20 | 168 | 0.56380 |
| Group: Age at onset | 2 | 0.25296 | 1.2163 | 20 | 168 | 0.24640 |

These are results from a MANOVA testing the multivariate association between the top 10 lipid-derived principal components and clinical/biological covariates. Pillai’s trace was used as the test statistic due to its robustness to violations of MANOVA assumptions. The model included main effects of Group, Age at Sampling, Age at Onset, Sex, BMI, and ALSFRS at sampling, as well as the interaction between Group and other variables. Degrees of freedom correspond to the multivariate test across 10 dependent variables (PC1 to PC10). The results indicate no influence of covariates associated with systematic multivariate variation in lipid composition.
