# Supplementary material for: Disruption of the angiopoietin-like system connects lipid homeostasis and hypothalamic dysfunction in ALS
Source: BMC Med. 2026 Mar 3;24:210. doi: 10.1186/s12916-026-04749-4 (PMC13064175; doi:10.1186/s12916-026-04749-4)
Supplement: Supplementary file 2 — Additional file 2. Figures S1–S6. Fig. S1 Reduced total cholesterol in mSOD1 patients. Fig. S2 Lipidome of ALS patients separate based on genetics. Fig. S3 Lack of association between serum ANGPTL4 and neuroimaging endophenotypes. Fig. S4 Altered expression levels of ANGPTLs in peripheral tissues. Fig. S5 The lipidome of SOD1 animals is different from their WT counterparts at the symptomatic stages. Fig. S6 Peripheral ANGPTL-4 correlates with hypothalamic AgRP. [file 12916_2026_4749_MOESM2_ESM.docx]

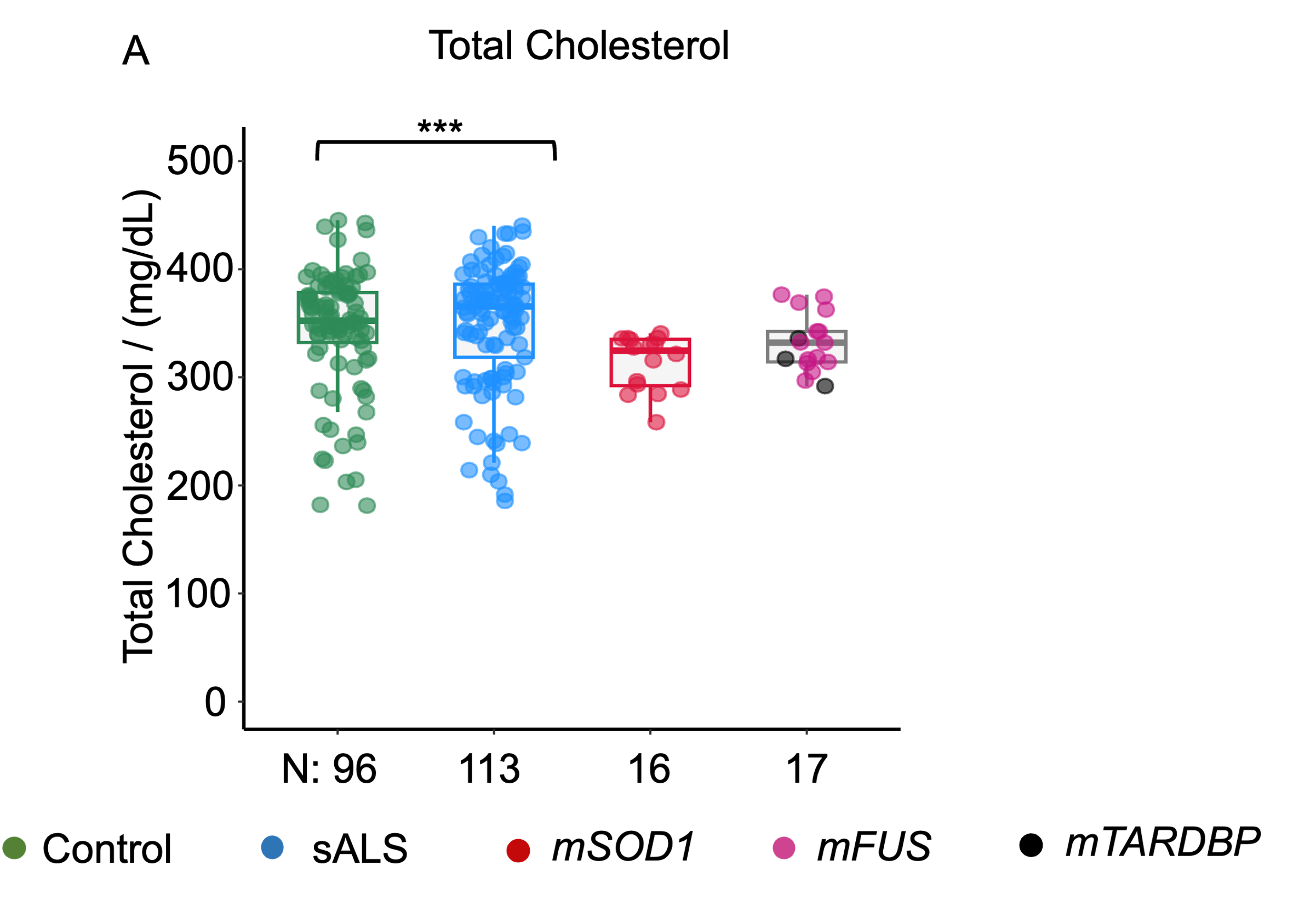


**Fig. S1 Reduced total cholesterol in mSOD1 patients**

Total cholesterol is decreased in SOD1 ALS patients compared to healthy controls. N is the number of patients per group for each assay. Box plot shows the median and interquartile range (IQR). Whiskers extend to the most extreme data point within 1.5 times the IQR. Individual data points are also shown. Statistical significance was determined by a two-way ANOVA, followed by Tukey's HSD post-hoc test for pairwise comparisons **P* < 0.05, ** *P* < 0.01, *** *P* < 0.001.


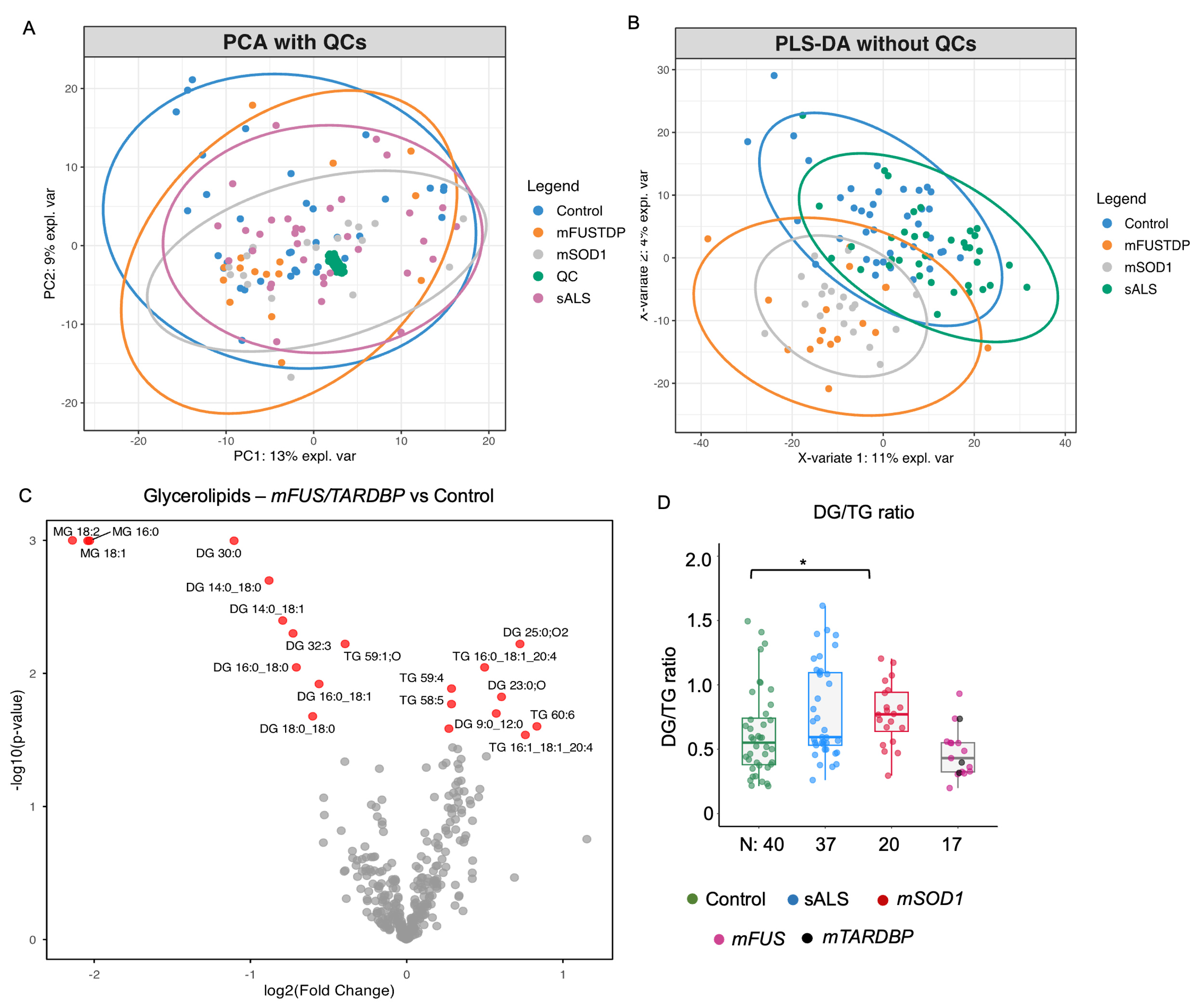


**Fig. S2** **Lipidome of ALS patients separate based on genetics**

Panel **A** shows the PCA plot with quality controls, to demonstrate technical quality. **B** shows the separation of different groups of ALS patients based on genetics and healthy controls. Ellipses indicate the 95% confidence region for each group's centroid, highlighting the separation and clustering of samples in the reduced dimensional space. Panel **C** represents a volcano plot showing the fold change on the x-axis and FDR adjusted p-values on the y-axis for all lipid species belonging to the superclass Glycerolipids (mainly composed of TG, DG and MG) for the *FUS/TARDBP* group of patients compared to healthy controls. **D** shows a significantly increased DG/TG ratio for all lipid species belonging to the subclass DG and TG in the *SOD1* group. N is the number of patients per group for each analysis. Box plot shows the median and interquartile range (IQR). Whiskers extend to the most extreme data point within 1.5 times the IQR. Individual data points are also shown. Statistical significance was determined by a two-way ANOVA, followed by Tukey's HSD post-hoc test for pairwise comparisons **P* < 0.05, ** *P* < 0.01, *** *P* < 0.001.

**
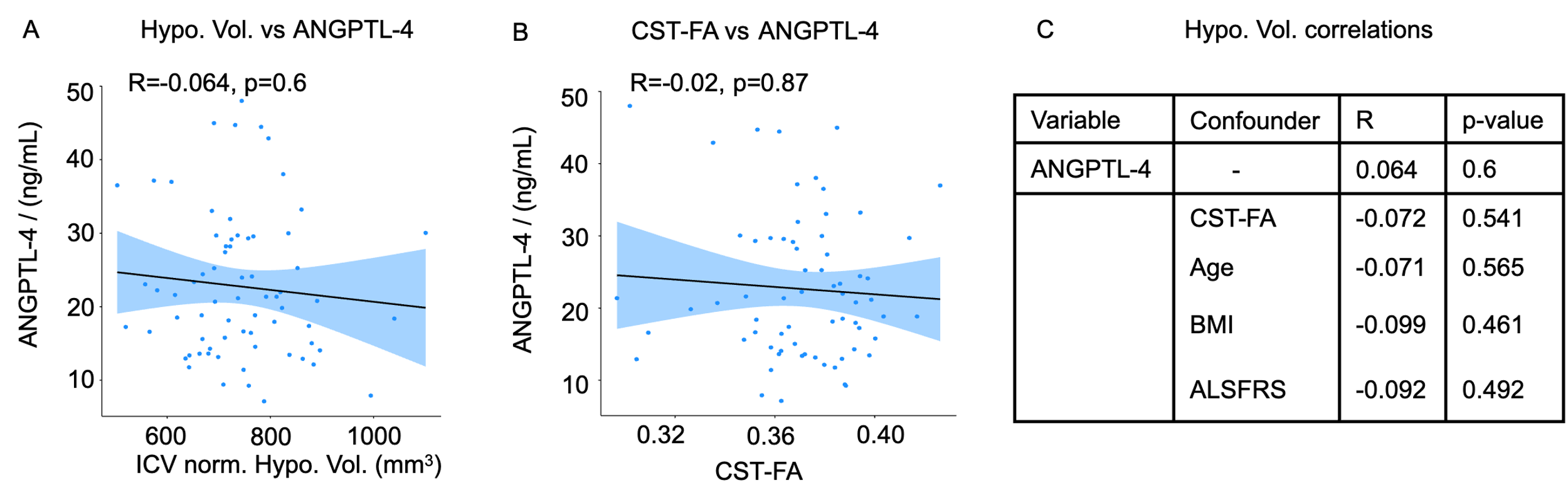
**

**Fig. S3** **Lack of association between serum ANGPTL4 and neuroimaging endophenotypes**

**A**. No correlation of ICV normalised hypothalamic volume with serum ANGPTL-4 levels; Spearman’s rho of -0.064 with a p-value of 0.6.

**B**. No correlation of CST-FA values with serum ANGPTL-3; Spearman’s rho of -0.02 with a p-value of 0.87.

**C.** No relationship between hypothalamic volume and serum ANGPTL-3 in the presence of confounders such as CST-FA, Age, BMI and ALSFRS as determined by multivariate correlation analysis. R is Spearman’s rho, and the p-value is adjusted for the false discovery rate by the Benjamini-Hochberg method.

**
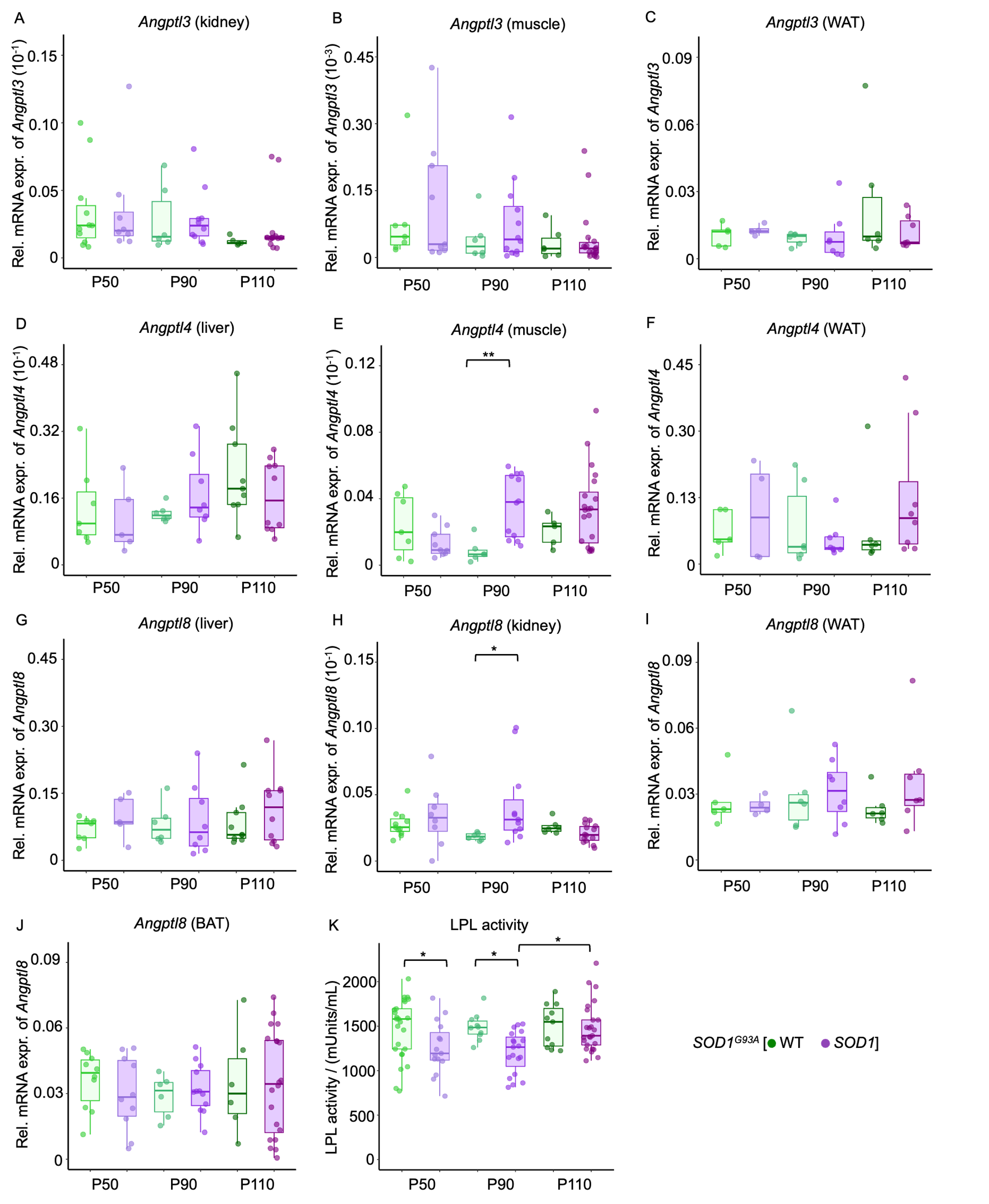
 Fig. S4** **Altered expression levels of ANGPTLs in peripheral tissues**

**A** to **J** show relative mRNA expression of the corresponding Angptl gene with Gapdh, as measured via qPCR analyses in *SOD1G93A* mice compared with their corresponding WT littermates. **K** shows the reduced activity of Lipoprotein lipase in *SOD* mice at pre- and early symptomatic stages, with a significant increase at the fully symptomatic stage. Box plots show the median and interquartile range (IQR). Whiskers extend to the most extreme data point within 1.5 times the IQR. Individual data points are also shown. Statistical significance was determined by a two-way ANOVA, followed by Tukey's HSD post-hoc test for pairwise comparisons **P* < 0.05, ** *P* < 0.01, *** *P* < 0.001. For SOD1G93A mice, P50 is the pre-symptomatic stage, P90 is the early symptomatic stage, and P110 is the fully symptomatic stage.

**
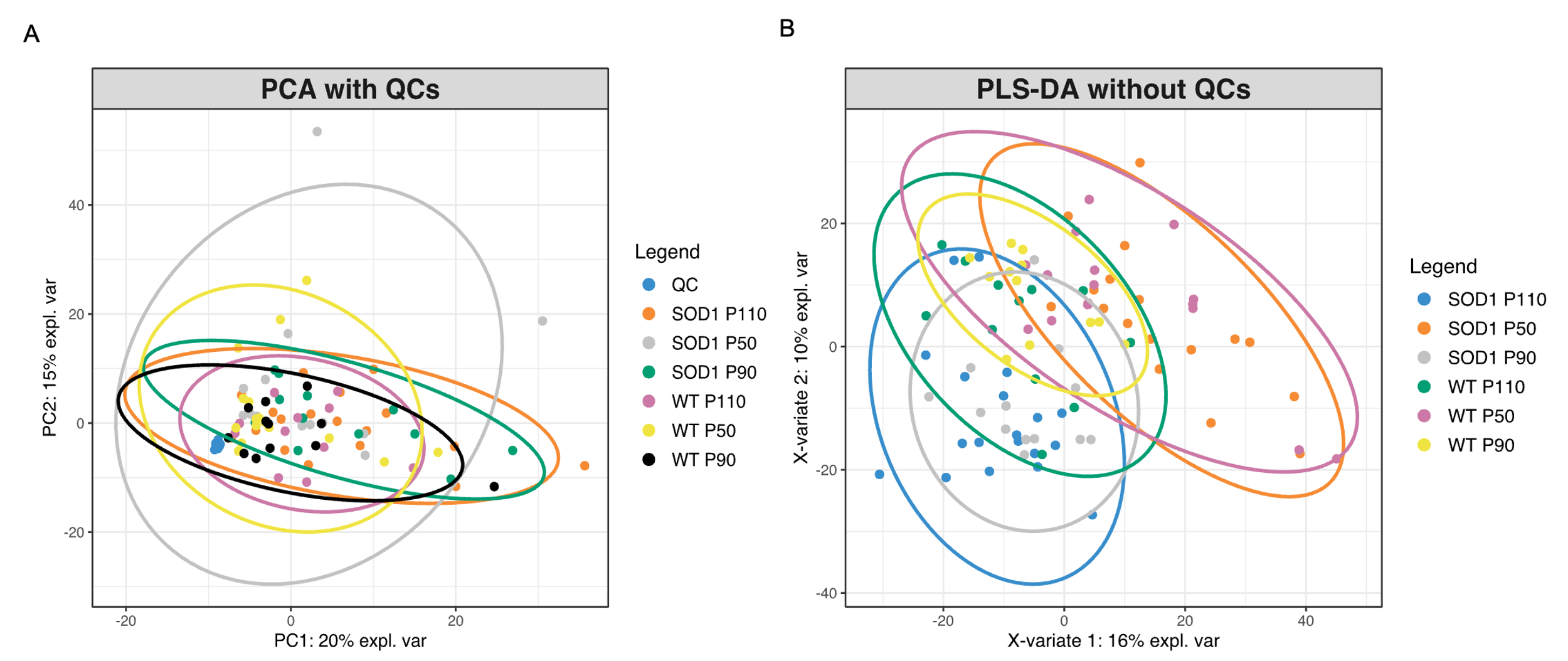
 Fig. S5** **The lipidome of SOD1 animals is different from their WT counterparts at the symptomatic stages**

Panel **A** shows the PCA plot with quality controls, to demonstrate technical quality. **B** shows the separation of WT and SOD1 mice at P50, P90 and P110, with a clear separation of the lipidome at P90 and P110. Ellipses indicate the 95% confidence region for each group's centroid, highlighting the separation and clustering of samples in the reduced dimensional space.

**
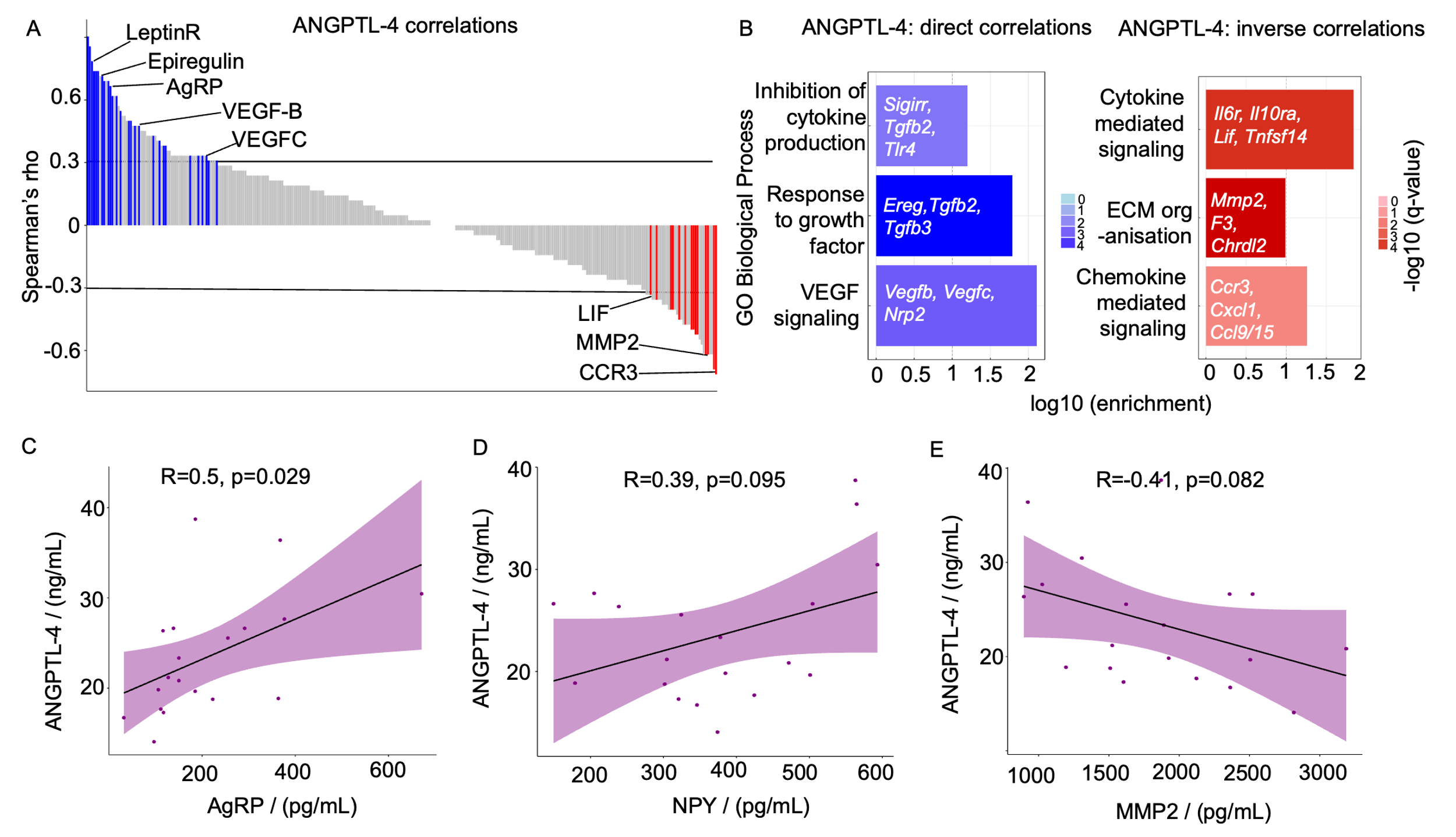
 Fig. S6 Peripheral ANGPTL-4 correlates with hypothalamic AgRP**

**A** shows the correlation of systemic ANGPTL-4 levels of *SOD1* mice with all the 308 proteins in the antibody array. All proteins correlating directly or inversely with an estimate of ± 0.30 and statistically significant are marked blue or red depending on the direct or inverse correlation estimates. **B** represents the selected gene ontology enrichment analysis of the proteins directly and inversely correlated with systemic ANGPTL-4, respectively. The enrichment for **B** was done via Stringdb against the gene set of Mus musculus, with enriched genes adjusted for FDR. **C** and **D** show a positive correlation and a trend towards correlation of systemic ANGPTL-4 with hypothalamic AgRP (R=0.5, *P*=0.029) and NPY (R=0.39, *P*=0.095), respectively, as measured by ELISA in a larger cohort (*n*=19) of *SOD1* P110 mice. **E** shows a trend towards inverse correlation of hypothalamic MMP2 with systemic ANGPTL-4 (R=-0.41, *P*=0.082) in *n*=19 *SOD1* P110 mice, thereby validating the results from the antibody array. For C to E, R is the Pearson’s correlation coefficient.
